# Development and Validation of Risk Prediction Models for Gestational Diabetes Mellitus Using Four Different Methods

**DOI:** 10.3390/metabo12111040

**Published:** 2022-10-29

**Authors:** Ning Wang, Haonan Guo, Yingyu Jing, Lin Song, Huan Chen, Mengjun Wang, Lei Gao, Lili Huang, Yanan Song, Bo Sun, Wei Cui, Jing Xu

**Affiliations:** 1 Department of Endocrinology, The Second Affiliated Hospital of Xi’an Jiaotong University, Xi’an 710004, China; 2 International Center for Obesity and Metabolic Disease Research of Xi’an Jiaotong University, Xi’an 710061, China; 3 Department of Endocrinology and Second Department of Geriatrics, The First Affiliated Hospital of Xi’an Jiaotong University, Xi’an 710061, China; 4 Department of Physiology and Pathophysiology, School of Basic Medical Sciences, Xi’an Jiaotong University Health Science Center, Xi’an 710061, China; 5 Department of Endocrinology, 521 Hospital of Norinco Group, Xi’an 710065, China; 6 Department of Medical Ultrasound, The Second Affiliated Hospital of Xi’an Jiaotong University, Xi’an 710004, China

**Keywords:** gestational diabetes mellitus, prediction models, risk factors, early pregnancy

## Abstract

Gestational diabetes mellitus (GDM), a common perinatal disease, is related to increased risks of maternal and neonatal adverse perinatal outcomes. We aimed to establish GDM risk prediction models that can be widely used in the first trimester using four different methods, including a score-scaled model derived from a meta-analysis using 42 studies, a logistic regression model, and two machine learning models (decision tree and random forest algorithms). The score-scaled model (seven variables) was established via a meta-analysis and a stratified cohort of 1075 Chinese pregnant women from the Northwest Women’s and Children’s Hospital (NWCH) and showed an area under the curve (AUC) of 0.772. The logistic regression model (seven variables) was established and validated using the above cohort and showed AUCs of 0.799 and 0.834 for the training and validation sets, respectively. Another two models were established using the decision tree (DT) and random forest (RF) algorithms and showed corresponding AUCs of 0.825 and 0.823 for the training set, and 0.816 and 0.827 for the validation set. The validation of the developed models suggested good performance in a cohort derived from another period. The score-scaled GDM prediction model, the logistic regression GDM prediction model, and the two machine learning GDM prediction models could be employed to identify pregnant women with a high risk of GDM using common clinical indicators, and interventions can be sought promptly.

## 1. Introduction

Gestational diabetes mellitus (GDM) is usually diagnosed in the second and third trimesters, and these pregnant women have no history of diabetes before pregnancy. GDM is characterized by hyperglycemia during pregnancy [1], which increases the risk of developing postpartum type 2 diabetes mellitus (T2DM) among pregnant women [2], along with premature delivery and clinical neonatal hypoglycemia [3]. However, the fetus of GDM women exposed to an intrauterine hyperglycemic environment from the beginning of the second trimester may lead to smaller fetuses [4] and abnormal abdominal circumference growth rates relative to pregnant women with normal glucose tolerance (NGT) [5].

The 75 g oral glucose tolerance test (OGTT) during the 24th–28th week of pregnancy is performed to diagnose GDM [1]. However, the fasting status and strict interval limitation for gestational age required for screening are unrealistic in some low-income areas in western China.

Some studies have employed the levels of angiopoietin-like protein 8, plasma fatty acid binding protein 4, and various adipokines [6,7,8] to predict GDM at the early stages of pregnancy but these have been difficult to popularize in clinical settings. Other common clinical indicators, including excessive weight gain during pregnancy, increased pre-pregnancy body mass index (pre-BMI) [9,10,11], advanced maternal age [12], accepted assisted reproductive technology (ART) treatment [13], family history of T2DM [14], and young menarche age are risk factors for GDM [15], which can be used as potential predictors of GDM development but show poor predictive powers as validated by our data. We screened several published GDM prediction models by literature review, and some of these have limited clinical utility due to the uncommon use of the included variables. Kang and colleagues [16] developed a model comprising several clinically uncommon variables, including the levels of HbA1c, IgA, triglycerides, and the percentage of B lymphocytes at the early stages of pregnancy. The variables from the model of Schoenaker and colleagues [17], including diet and exercise, are difficult to measure in clinical settings. Finally, the GDM prediction model of Sweeting and colleagues [18], which targets the Asian population in different regions, has not been validated in China. Additionally, we validated three published GDM prediction models [19,20,21] using data from a cohort obtained in May 2021 from the Northwest Women’s and Children’s Hospital (NWCH), and these showed limited performances (Supplementary Table S10).

Therefore, we combined these risk factors to establish individualized GDM prediction models to screen women at a high risk of developing GDM during the early stages of pregnancy and guide the implementation of prevention strategies.

## 2. Materials and Methods

### 2.1. Data Sources

In the beginning, we screened data of 1075 pregnant women from a retrospective cohort at the Department of Obstetrics at NWCH between November 2019 and March 2020. To validate the performances of the established models, we collected data from the NWCH cohort in May 2021. We obtained data from the routine gestational care visit. Women carrying a full-term fetus and relatively complete pregnancy data were included. Women with diabetes before pregnancy who met the criterion for OGTT and were diagnosed with T2DM or other metabolic diseases were excluded. Finally, 1075 pregnant women were included for further analyses.

### 2.2. Outcomes

GDM or NGT was diagnosed according to the IADPSG criteria [22] between the 24th and 28th gestational week. All the included pregnant women in this study did not undergo OGTT in the 1st trimester and showed a corresponding fasting blood glucose (FBG) < 5.1 mmol/L.

### 2.3. Clinical Measurements and Definitions

We calculated pre-BMI using the recorded weight (maternal weight before and during pregnancy were recorded in the electronic medical records) divided by the height squared (m^2^) at the 1st gestational care visit and stratified based on pre-BMI using the criteria specific for Chinese adults [23] as follows: ≤23.9 kg/m^2^ as normal, 24–27.9 kg/m^2^ as overweight, and ≥28 kg/m^2^ as obese women. We stratified maternal age into <30 years (yr), 30–34 yr, 35–39 yr, and ≥40 yr categories. The 1st-trimester gestational weight gain (GWG) was calculated as the maternal weight at the beginning of the 2nd trimester minus the pre-pregnancy weight, and the “above the recommended GWG” was defined based on the Institute of Medicine guideline (IOM) recommendations [24]. A weight gain of more than 2 kg in the 1st trimester was considered excessive for all the pre-BMI stratification groups. Age at menarche was grouped into <11 yr and ≥11 yr categories, as existing literature suggests that menarche age < 11 yr is a risk factor for metabolic diseases and fetal overgrowth during pregnancy [25,26,27]. FBG in the 1st trimester was stratified into two groups using a 5.0 mmol/L cut-off value as the recommended FBG level during early pregnancy [28]. These stratifications were based on clinical knowledge and practice guidelines. The positive status of relative thyroid antibodies in this study was defined as the positive status of TPO-Ab/Tg-Ab in the 1st trimester.

### 2.4. Data Collection and Detection of Plasma Biochemical Parameters

The questionnaire survey for participants included general information on medical history, T2DM family history, reproductive history, and age at menarche. All laboratory tests were performed by standard methods at a certified laboratory. The glucose oxidase method was used to test FBG levels, with an intra-and inter-assay variation factor of 2.1% and 2.6%, respectively. The enzyme-catalyzed method was employed to obtain the plasma lipid profiles (triacylglycerol (TG), total cholesterol (CHO), low-density lipoprotein cholesterol (LDL-C), and high-density lipoprotein cholesterol (HDL-C)). Plasma thyroid function was tested using commercially available kits (FT3 (R-A-03-01, 1–81 pmol/L), FT4 (R-A-04-01, 3–200 pmol/L), TSH (R-A-05-01, 0–50 uIU/mL), TG-Ab (R-A-07-01, 30–3000 IU/mL), and TPOAb (R-A-08-01, 10–1000 IU/mL) 3V Bioengineering, Shandong, China). The indexes of liver function (aspartate aminotransferase (AST), alanine aminotransferase (ALT), total protein, albumin, and globuli), vitamin B12, and ferritin levels were tested using the reagents manufactured by Shandong 3V Bioengineering Company, Weifang China, on the Hitachi 7600 automatic 49 biochemical analyzer platform.

### 2.5. Derivation Cohort for the Score-Scaled GDM Risk Prediction Model

**Figure 1:**
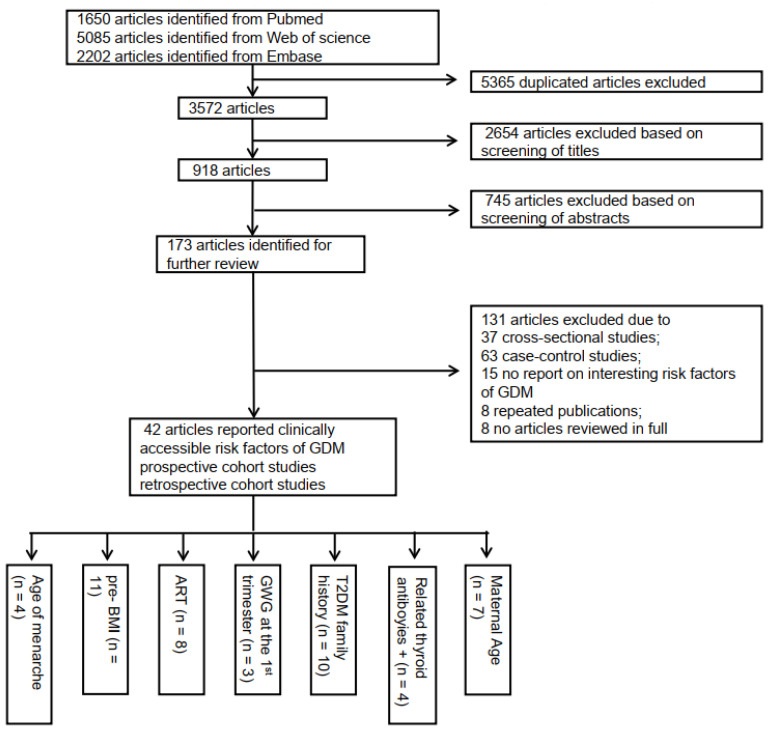
Flow diagram outlining the literature search and selection based on risk factors of GDM development in pregnant women at early pregnancy.

The meta-analysis for the derived cohort, comprising cohorts from 42 studies (12 prospective and 30 retrospective cohorts), was performed with a PROSPERO ID CRD42022302930. We searched for articles that were published in electronic databases, including MEDLINE, Embase, and Web of Science until the end of 10 July 2021, using a merged method consisting of MeSH headings search strategy and the following terms: “Diabetes, Gestational”, “Risk Factors”, and “Cohort Studies.” Pregnant women were from Asia (China, Iran, Israel, Japan, Korea, and Malaysia), Europe (Finland, Italy, Spain, Sweden, and the UK), the Americas (U.S. and Peru), and Australia, and three international multi-center cohorts were included. Odds ratios (ORs) and corresponding 95% CIs (confidence intervals) were estimated for the risk factors extracted from these articles. We used the Newcastle Ottawa Scale to evaluate the quality of the included studies. We used Endnote to screen the titles and abstracts after removing duplicate reports; WN and GHN screened independently. In cases of differences, a third investigator (JYY) was involved for discussion and resolution. Full texts of the screened studies were evaluated by WN and GHN according to the set criteria, and any disagreement was resolved unanimously with the participation of the third investigator (JYY). Further, the data were extracted and checked by WN, GHN, and JYY. Figure 1 presents the flow chart of the research report selection method. Supplementary Tables S1 and S2 list the keywords, derivative words, and retrieval strategy.

### 2.6. Statistical Analysis

Continuous variables showing normal distribution were presented as means ± standard deviation (SD). Differences between the NGT and GDM groups were compared using the *t*-test; the Chi-squared test was utilized for categorical variables.

#### 2.6.1. Meta-Analysis

ORs with 95% CIs of all risk factors for GDM were extracted and analyzed using random-effects or fixed-effects models based on their heterogeneity. According to the statistical estimate of the sample size, the inverse of the variance of the OR was the corresponding weight of the study [29]. The heterogeneity of each study was assessed and measured using I^2^; when I^2^ was >50%, the random-effects model was used; otherwise, the fixed-effects model was chosen [30]. If risk factors showed significant heterogeneity (I^2^ > 50%), sensitivity analyses were conducted by omitting a single study to measure the robustness of the results [31]. Funnel plots were drawn to measure the publication bias. *p*-value < 0.05 in a two-sided test suggested statistical significance. We used the Review Manager software version 5.0 for the meta-analysis.

#### 2.6.2. Multiple Imputations

Multiple imputations with chained equations were employed to replace the missing values for vitamin B12, ferritin, CHO, TG, LDL-C, and HDL-C. The number of all missing values was within 20% of the total. Five estimation models were used based on the sample size and the capacity of the software (Jupyter Notebook 6.4.5, python 3.9.7).

#### 2.6.3. The Logistic Regression Modeling Strategy

We divide pregnant women into the training (*n* = 765) and validation (*n* = 310) sets randomly. Variables were screened by Lasso regression, and these in the model were determined by cross-verification Lasso logistic regression (3 folds, seed 123), which, with the largest lambda for MSPE, are within one standard error (STATA 15.0). The regression modeling strategy employed multivariate logistic regression (SPSS 22.0). The nomograph was constructed and AUCs were measured using STATA (version 15.0).

#### 2.6.4. The Machine Learning (ML) Algorithms

The original data consisted of 722 NGT and 352 GDM women. In order to improve the performance of the ML, a combination of over-sampling by SMOTE (synthetic minority oversampling technique) for the minority class and random under-sampling for the majority class is used to balance the dataset. The DT and RF algorithms were used to establish GDM prediction models using Jupyter Notebook (Anaconda) 6.4.5. All graphs for ML models were plotted using Graphviz 2.38.

### 2.7. Development and Validation of the Models

#### 2.7.1. The Score-Scaled GDM Risk Prediction Model

Considering the practicality and clinical utility of the prediction model, we only extracted the pooled ORs and their 95% CIs for risk factors with statistically significant differences and selected the appropriate ORs for sensitivity analyses, based on the criteria proposed by Sullivan and colleagues [32]. Scores of risk factors were calculated by multiplying the β-coefficient by 10 and rounding it off. The total score was the sum of each risk factor. The data of 1075 pregnant women from November 2019 to March 2020 were used for the risk stratification, and validation was performed using the data of 210 pregnant women from the May 2021 cohort. The total score was calculated for each pregnant woman based on the variables in the model. The occurrence of GDM in the 2nd trimester was the outcome of assessing the AUC. According to the optimal cut-off value and the median score of the two intervals [33], pregnant women were divided into 4 groups, namely relatively low, moderate, high, and very high, to analyze the differences in the proportion of outcomes.

#### 2.7.2. Logistic Regression Analysis for GDM Risk Prediction Model

Variables were screened by Lasso regression analysis, and cross-verification was performed. Multivariate logistic regression (backward variable selection) was conducted using the training set. Equations and nomographs were used for the popularization of the prediction model. The validation set comprised 310 women, and the AUC reflected an estimate of the average optimal model’s predictive accuracy, which also quantified the level of agreement between the predicted probabilities and the actual incidence of GDM. The calibration curve was plotted to evaluate the association between the probability estimated using the logistic regression model and the observed GDM rate in training/validation sets. The Hosmer–Lemeshow (H-L) test was conducted to determine the differences between the predicted and the true values. The decision curve showed the net return from the model. The model was further validated using data from 210 pregnant women from the May 2021 cohort.

#### 2.7.3. ML Prediction Models

Two ML algorithms, namely DT and RF, were employed. DT is a tree structure consisting of a single root node and several internal nodes and leaf nodes. The starting node is the root and the path from the root to the leaf node represents the classification processes of DT. CART algorithms based on the Gini coefficients were used. RF is an integrated algorithm layered on top of multiple DT classifiers. Each DT in the RF is randomly constructed and controlled by several selected characteristic variables. The learning process includes bagging and random feature selection.

To achieve a better prediction performance by ML algorithms, we used the Random Under-Sampling and Synthetic Minority Over-Sampling Technique (SMOTE) to balance the data. Data were randomly divided into training (70%) and validation (30%) sets for DT and RF algorithms, respectively. The final classification results mostly contained DTs. Indicators such as the AUC-ROC curve, precision, recall, Fi-score, accuracy, and specificity were employed to measure the performance of these ML prediction models.

## 3. Results

### 3.1. The Score-Scaled GDM Risk Prediction Model

#### 3.1.1. Derivation Cohort

The patients in the derivation cohort were from 42 different studies, and the period ranged from 1 to 19 years. Supplementary Table S3 lists the characteristics of these studies. The qualities of these studies were measured using the Newcastle-Ottawa scales by SL and CH independently, and the scores ranged from 6 to 9 (Supplementary Table S4).

**Figure 2:**
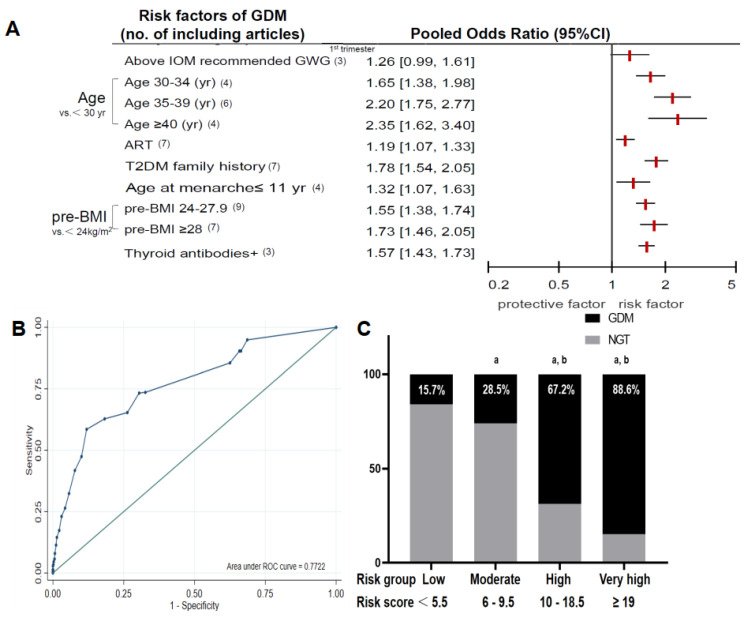
The score-scaled GDM prediction model. (**A**) Pooled ORs and their corresponding 95% CIs for risk factors in the score-scaled GDM risk prediction model. Estimates were derived from the fully adjusted models in each included analysis. Red squares and horizontal bars represent the overall estimates and 95% CIs, respectively. (**B**) ROC curve for the validation cohort based on the score-scaled risk prediction model. (**C**) Prevalence of GDM in the four groups of the validation cohort. Pairwise comparisons were adjusted using the Bonferroni correction method. ^a^ different relative to the low-risk group by the Chi-squared test. ^b^ different as compared to the moderate risk group by the Chi-squared test.

Seven reasonable risk factors were selected following the meta-analysis. Pooled ORs and their corresponding 95% CIs are shown in Figure 2A (the detailed data, forest plots, sensitivity analyses, and funnel figures of these factors are provided in Supplementary Table S5 and Supplementary Figures S1–S9). Finally, the GDM risk prediction model was established with age (<30 yr scores 0, 30–34 yr scores 5.0, 35–39 yr scores 8.0, and ≥40 yr scored 9.0), BMI (<24.0 kg/m^2^ scores 0, 24–27.9 kg/m^2^ scores 4.0, and ≥28 kg/m^2^ scores 5.0), T2DM family history (no T2DM family history scores 0 and have T2DM family history scores 6.0), age at menarche (0 if age at menarche > 11 and 3.0 if ≤11 years), acceptance of ART treatment (0 if no and 2.0 if yes), the positivity of thyroid-related antibodies (0 if no and 5.0 if yes), and IOM above the recommended GWG in the first trimester (0 if no and 2.0 if yes) (shown in Table 1).

#### 3.1.2. Validation Cohort

The clinical and serological indicators in the first trimester in the GDM and NGT groups are shown in Supplementary Table S6. Compared to the NGT group, women in the GDM group were older. The higher proportions of pregnant women with T2DM family history, higher pre-BMI and FBG levels, and larger proportion of women in the age at menarche ≤ 11 yr, acceptance of ART treatment, the positivity of thyroid-related antibodies, IOM above the recommended GWG in the first trimester, and a history of macrosomia were present in the GDM group relative to the NGT group (all *p* < 0.05). The model showed an AUC of 0.772 (95% CI 0.742–0.803) (Figure 2B). The optimal cut-off score was 9.5 with the maximum corresponding Youden index (Supplementary Table S7). Based on this model, 1075 pregnant women were further classified into four groups based on risk scores as follows: <5.5 as low (*n* = 580), 6–9.5 as moderate (*n* = 203), 10–18.5 as high (*n* = 259), and ≥19 as very high (n = 33) risk levels. The number of included pregnant women who developed GDM in the second trimester was 93 (15.7%) in the low group, 53 (28.5%) in the moderate group, 178 (67.2%) in the high group, and 28 (88.6%) in the very high group (Figure 2C). Significant statistical differences were found in the pairwise comparisons among the four groups (except for the high and the very high-risk groups).

### 3.2. Logistic Regression Analysis for the GDM Risk Prediction Model

Table 2 shows the comparison of clinical characteristics between the training cohort (*n* = 765) and validation cohort 1 (*n* = 310) and validation cohort 2 (*n* = 210).

#### 3.2.1. Training Set

**Figure 3:**
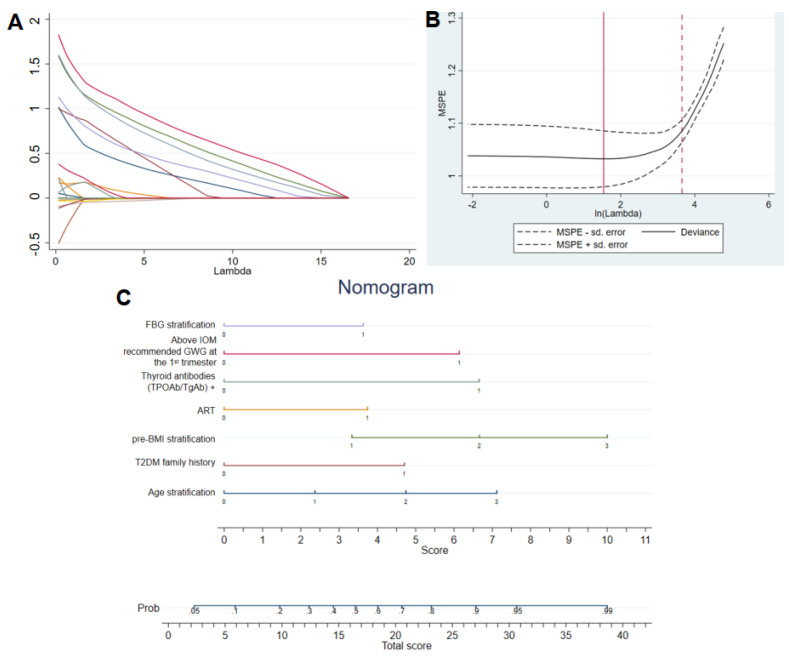
The logistic regression analysis for GDM risk prediction model. (**A**) Lasso-logistic graph. (**B**) Cross-validation of lasso-logistic regression results. (**C**) Nomogram to predict the probability of developing GDM at the first trimester among pregnant women. The total score calculated by summing the scores of FBG stratification, GWG at the first trimester, the positive status of thyroid antibodies, acceptance for ART treatment, pre-BMI stratification, T2DM family history, and age stratification.

Seven variables were included in the model by cross-validated lasso-logistic regression (Figure 3A,B). Multivariate logistic regression was used for the calculation and these results are shown in Table 3. The nomogram was used to predict the incidence of GDM in pregnant women in the first trimester (Figure 3C). The predicted GDM risk was estimated using the following equation:

$$P=\frac{1}{1+exp.\left(-x\right)},\;X=-3.417+0.492\;\mathrm{Maternal}\;\mathrm{Age}\;30–34y/0.984\;\mathrm{Maternal}\;\mathrm{Age}\;35–39y/1.492\;\mathrm{Maternal}\;\mathrm{Age}\;\geq \;40y+0.976\;T2\mathrm{DM}\;family\;history+0.691\;\mathrm{pre}\text{-}\mathrm{BMI}\;24–27.9\;\mathrm{kg}/m^{2}/1.382\;\mathrm{pre}\text{-}\mathrm{BMI}\;\geq \;28\;\mathrm{kg}/m^{2}+0.776\;\mathrm{ART}\;+1.381\;\mathrm{Thyroid}\;\mathrm{antibodies}\;\mathrm{positive}+1.273\;\mathrm{Above}\;\mathrm{IOM}\;\mathrm{recommended}\;\mathrm{GWG}\;\mathrm{at}\;\mathrm{the}\;1\mathrm{st}\;\mathrm{trimester}+0.753\;\mathrm{FBG}\;\geq \;5.0\;\mathrm{mmol}/L$$

#### 3.2.2. Discriminant Analysis

**Figure 4:**
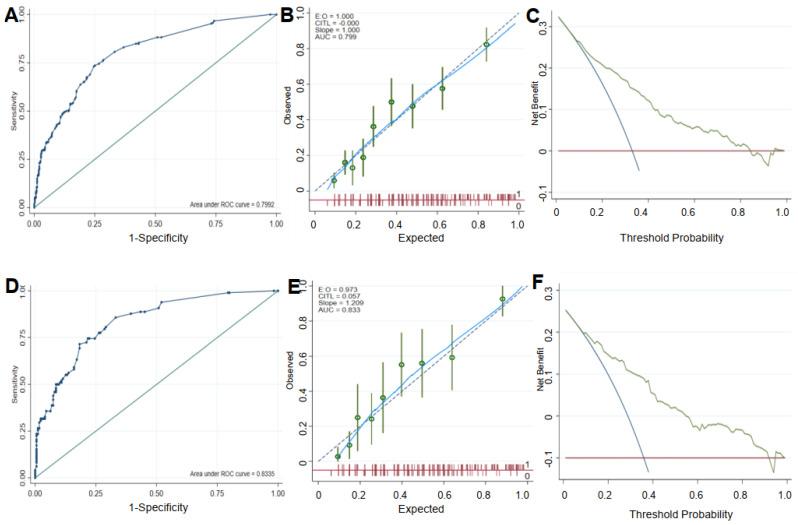
Discriminant results. ROC curve graphs for the training set (**A**) and the validation set (**D**). The calibration curve for the training set (**B**) and the validation set (**E**). The decision curve analysis for the training set (**C**) and the validation set (**F**).

The AUC of the ROC curve was 0.799 (95% CI 0.763–0.836) in the training set and 0.834 (95% CI 0.785–0.882) in the validation set. The calibration curve of the training and validation sets, showing the association between the probability of GDM predicted using the model and the observed GDM rate, suggested consistency between the two. The decision curves for the training and validation sets suggested a net benefit without increasing the number of false positives (Figure 4). The *p*-value for the H-L tests was 0.374 in the training set and 0.530 in the validation set, indicating that the logistic regression analysis prediction model had high consistency.

### 3.3. Comparison of the Two Prediction Models

The Net Reclassification Index (NRI) and Integrated Discrimination Improvement (IDI) were focused on comparisons of the two prediction models at a certain truncation value. In this study, we compared the score-scaled GDM risk prediction model (model 1) with the logistic regression analysis for the GDM risk prediction model (model 2). The results showed positive improvements in model 2, with an NRI of 0.208 and IDI of 0.045 (Supplementary Table S8), implying that model 2 had better predictive power than model 1.

### 3.4. ML Models for GDM Prediction

The data of AUC, precision, recall, Fi-score, accuracy, and specificity of training and validation sets for the DT and RF models are shown in Supplementary Table S9. The ROC curves, tree structures, and feature importance curves for DT and RF models are illustrated in Supplementary Figures S10–S13.

### 3.5. Validation of the Established Models

We used data from the NWCH cohort of May 2021 to further validate the performance of the developed models, and the results suggested a good performance. The AUC was 0.769 (0.681–0.858) for the score-scaled model, 0.841 (0.736–0.891) for the logistic regression model, 0.777 (0.726–0.829) for the DT model, and 0.740 (0.684–0.795) for the RF model (Supplementary Table S10).

## 4. Discussion

We established GDM prediction models using meta-analysis, logistic regression, and two ML algorithms. For the score-scaled model, the following risk factors were screened for meta-analysis: Maternal age, pre-BMI, GWG in the first trimester, age at menarche, ART, T2DM family history, and positivity for thyroid-related antibodies (TPOAb and TgAb). Data for risk stratification was collected from 1075 pregnant women who carried the fetus to full-term with relatively complete clinical data from NWCH; the AUC was 0.722. Statistically, differences were present among the four risk groups divided at the cut-off point of 9.5 (except for the high and very high-risk groups). For the logistic regression analysis model, the variables included maternal age, pre-BMI, GWG in the first trimester, acceptance of ART treatment, T2DM family history, the positivity of thyroid-related antibodies, and FBG. The training and validation sets showed AUCs of 0.799 (95% CI 0.763–0.836) and 0.834 (95% CI 0.785–0.882), respectively. The prediction models developed using two ML algorithms (DT and RF) comprised all the above-mentioned risk factors and showed relatively reasonable prediction performances in both the training and validation sets. All the models were validated using data from the cohort collected in May 2021 at NCWH and showed good performances.

For the correlation of the risk factors and GDM, a dose–response association from a meta-analysis [12] showed a linear relationship between the risk of developing GDM and advanced maternal age, with the same age stratification as in our study. Overweight/obesity before pregnancy, excess GWG [9,10,11], and younger menarche age [14] are risk factors for GDM and may be mediated by the status of insulin resistance. T2DM is a polygenic inherited disease with overlapping susceptibility genes as in GDM [15]. Elevated FBG levels in the first trimester were associated with GDM [34]; however, the majority of studies show inconsistent cut-off values [35,36]. Some particular ART procedures are related to the increased incidences of GDM [13], which may be caused by advanced maternal age and underlying maternal subfertility-related diseases. The positivity of thyroid-related antibodies (TPOAb or TgAb) was associated with the risk of developing GDM [37]. Increased TPOAb/TgAb in the peripheral blood of patients with Hashimoto thyroiditis can interfere with the homeostasis of β-cells by reducing the number of CD19^+^CD24^hi^CD38^hi^ Breg cells and releasing inflammatory factors in peripheral blood, leading to insulin resistance [38,39]. However, any single clinical indicator is insufficient to predict the incidence of developing GDM accurately. The integration of published risk factors is more reliable for the early diagnosis of GDM.

Existing published predictors of GDM including angiopoietin-like protein 8, plasma fatty acid binding protein 4, and various adipokines are not popular in clinical practice [6,7,8]. Additionally, the data of some GDM prediction models [40,41] were from small cross-sectional and case-control studies, which is not conducive to application in the cohort of pregnant women. For a GDM prediction model that was developed in a retrospective study comprising 580,000 electronic medical records of Israeli pregnant women, the AUC was 0.85 after including all GDM-related variables, while the AUC of the questionnaire prediction model using only nine GDM-related variables was 0.80 [42]. However, the variables in these models were obtained in the 20th gestational week, and thus, GDM could not be predicted and intervened in the early stages of pregnancy. Our score-scaled GDM prediction model was developed using a meta-analysis of 42 high-quality cohort studies, which significantly improved the statistical performance and ease of calculation. The risk factors in our GDM prediction models are easily accessible from the clinical work in the first trimester. These advantages make our model more convenient and easier to use in clinical settings.

To our knowledge, a few published score-scaled GDM risk prediction models have been previously derived from a meta-analysis, which can be used in the first trimester in clinical settings. Several GDM-related risk factors, which were published previously, are not included in our meta-analysis, because of the differential diagnostic criteria for GDM.

The score-scaled GDM risk prediction model has numerous advantages, including being common across clinical settings and ease of generalization in underdeveloped areas. Notably, the model was established based on pregnant women who were >18 years old and is well-suited to this group. Moreover, two different criteria for the classification of women’s pre-BMI in the included studies were employed: (1) Among whites, the criteria were 18.5–24.9 kg/m^2^ for normal, 25.0–29.9 kg/m^2^ for overweight, and 30.0 kg/m^2^ for obese according to World Health Organization guidelines [43] and (2) in Chinese women, the criteria was 18.5–23.9 kg/m^2^ for normal, 24.0–27.9 kg/m^2^ for overweight, and above 28.0 kg/m^2^ for obese [44] according to the criteria recommended by the Working Group on Obesity in China, 2003.

The score-scaled GDM risk prediction model did not include FBG in the first trimester, owing to the inconsistency in the gestational week for FBG detection and the different cut-off values. Thus, the results could not be merged in the meta-analysis. However, in the logistic regression analysis model, the FBG level in the first trimester was a continuous variable, which enabled its stratification according to a published study [28].

For the logistic regression analysis model, logarithmic equations, a relatively tedious process, were employed to measure the probability of developing GDM. The corresponding nomogram could roughly estimate the probability of developing GDM; however, these results showed limited accuracy. Moreover, it may be unrealistic in some low-income areas, such as in western China, as it requires pregnant women to visit the hospital in the fasting state in the first trimester for assessing FBG levels.

Due to the embedded feature selection methods, limited large-scale extrapolation is inevitable for published and ML prediction models in this study [42,45]. Therefore, the ML algorithms in our study, as a method to establish the prediction models, are not recommended for clinical use. The published ML algorithm models comprise serological indicators, including HbA1c and TG, which are relatively hard to obtain in the early stages of pregnancy [42,45].

Modifiable risk factors in prediction models are necessary to identify and assess whether the relationship is causal, particularly important for prevention. In this study, GWG in the first trimester was identified as the modifiable factor, and thus, our prediction models can be used to evaluate the remission of incidence for developing GDM after an early intervention; however, this possibility needs to be tested further. 

Our study has some limitations. The data were extracted from a large tertiary institution, with relatively high-risk GDM incidence as compared to that in community hospitals or remote mountainous areas. As this was a single-center study, the cohort may not represent the diverse ethnic populations in China. As it was a retrospective study, we could not collect records of detailed information on diet and exercise, which may interfere with the basal metabolic rates and influence GWG, potentially mediating the association between GWG and GDM. Moreover, the first antenatal care visit of pregnant women is usually in the 12th gestational week, with potential recall bias in pre-pregnancy weight for calculating the pre-BMI. Finally, the prediction models included thyroid function indicators during the first trimester, a characteristic highlight of our study. Although the thyroid function test is becoming increasingly common in China during a regular prenatal visit, extensive testing of thyroid function in pregnant women remains difficult to implement in some remote areas, especially in western China, and a major limiting factor for the application of our prediction models in clinical settings.

## 5. Conclusions

We developed and validated GDM prediction models based on a meta-analysis, logistic regression, and two ML algorithms, which can be used to predict the incidence of GDM in the first trimester. The calculation method for the score-scaled GDM prediction model was relatively simple but showed lower prediction accuracy as compared to other models. The computational formula of the logistic regression GDM model was relatively complex but showed higher accuracy. The ML models exhibited the highest accuracy, but these are hard to implement in clinical practice. Clinicians can choose an appropriate prediction model based on the variables according to local settings. The predictive tools make it easy to identify women at high risk of developing GDM.

## Figures and Tables

**Table 1:** The score-scaled GDM risk prediction model.

Risk Factors for GDM	Category	Scores
Maternal age (years) *	<30	0
	30–34	5
	35–39	8
	≥40	9
T2DM family history	no	0
	yes	6
pre–BMI (kg/m^2^) **	<24	0
	24–27.9	4
	≥28	5
Age at menarche (year)	>11	0
	≤11	3
ART	no	0
	yes	2
The positive of related thyroid antibodies ***	no	0
	yes	5
Above IOM recommended GWG at the 1st trimester	no	0
	yes	2

GDM, gestational diabetes mellites; T2DM, type 2 diabetes mellites; pre-BMI, pregestational body mass index; ART, assisted reproductive technology; GWG, gestational weight gain. * Pregnant women in derivation and validation cohorts aged 18–45 years old. ** pre-BMI was categorized as <25.0, 25.0–29.9, and ≥30.0 kg/m^2^ in white patients and <24.0, 24.0–27.9, and ≥28.0 kg/m^2^ in Asians. *** Thyroid antibodies related to thyroid peroxidase antibodies (TPOAb) and the anti-thyroid peroxidase antibody (TgAb). *p* value < 0.05 is considered statistically significant.

**Table 2:** The clinical characteristics of training and validation cohorts.

Variables	Training Cohort (n = 765)	Validation Cohort 1 (n = 310)	Validation Cohort 2 (n = 210)
GDM	246 (32.2)	106 (34.2)	39(18.5)
Maternal age	31.77 ± 4.14	31.5 ± 4.03	31.24 ± 4.17
T2DM family history	70 (9.2)	33 (10.6)	15(7.1)
pre-BMI	21.97 ± 3.34	22.07 ± 2.97	21.4 ± 3.12
Age at menarche ≤ 11 yr	66 (8.6)	22 (7.1)	4(1.9)
ART	53 (6.9)	31 (10)	4(1.9)
Thyroid antibodies + (TPOAb/TgAb)	115 (15.0)	53 (17.1)	16(7.6)
Above IOM recommended GWG at the 1st trimester	134 (17.5)	57 (18.4)	34(16.1)
History of macrosomia	28 (3.7)	11 (3.5)	7(3.3)
Parity	1.50 ± 0.60	1.49 ± 0.65	1.22 ± 0.71
Vitamin B12 (pg/mL)	64.33 ± 7.34	65.36 ± 7.72	61.35 ± 6.51
Ferritin (ng/mL)	46.80 ± 5.57	47.03 ± 5.79	42.24 ± 4.63
Total protein (g/L)	69.05 ± 4.13	70.18 ± 3.88	65.43 ± 3.47
Albumin (g/L)	40.15 ± 2.31	40.0 ± 2.56	44.34 ± 3.27
Globulin (g/L)	29.90 ± 3.31	30.18 ± 3.25	31.58 ± 2.64
ALT (U/L)	17.11 ± 10.78	18.81 ± 11.83	17.31 ± 10.81
AST (U/L)	19.40 ± 9.38	18.66 ± 6.62	19.72 ± 7.24
CHO (mmol/L)	4.13 ± 0.73	4.16 ± 0.89	4.61 ± 0.63
TG (mmol/L)	1.51 ± 0.66	1.50 ± 0.75	1.60 ± 0.69
HDL-C (mmol/L)	1.67 ± 0.29	1.62 ± 0.27	1.80 ± 0.31
LDL-C (mmol/L)	2.31 ± 0.60	2.35 ± 0.58	2.43 ± 0.52
FBG (mmol/L)	5.04 ± 0.44	5.01 ± 0.41	4.88 ± 0.49

ALT, glutamic-pyruvic transaminase; AST, glutamic oxalacetic transaminase; CHO, total cholesterol; TG, triglyceride; HDL-C, high-density lipoprotein cholesterol; LDL-C, low-density lipoprotein cholesterol; FBG, fasting blood glucose. *p* value < 0.05 is considered statistically significant. Validation cohort 1: a cohort comprising pregnant women at NWCH enrolled from Nov. 2019 to Mar. 2020. Validation cohort 2: a cohort of pregnant women at NWCH enrolled in May 2021.

**Table 3:** The multivariable logistic regression analysis in the training cohort.

	B	S.E.	Wald	P	OR (95%CI)
Age stratification	0.492	0.131	14.202	<0.001	1.636 (1.266–2.113)
T2DM family history	0.976	0.307	10.120	0.001	2.653 (1.454–4.838)
pre-BMI stratification	0.691	0.167	17.153	<0.001	1.996 (1.439–2.769)
ART	0.776	0.381	4.154	0.042	2.173 (1.030–4.585)
Thyroid antibodies + (TPOAb/TgAb)	1.381	0.269	26.423	<0.001	3.979 (2.350–6.737)
Above IOM recommended GWG at the 1st trimester	1.273	0.239	28.470	<0.001	3.573 (2.238–5.703)
FBG stratification	0.753	0.204	13.625	<0.001	2.124 (1.424–3.169)
Constant	−3.417	0.356	92.142	<0.001	0.033

*p* value < 0.05 is considered statistically significant.

## Data Availability

Data supporting the reported results are stored at Xi’an Jiaotong University Second Affiliated Hospital.

## Supplementary Materials

The following supporting information can be downloaded at: https://www.mdpi.com/article/10.3390/metabo12111040/s1. Table S1. Keywords and derivative words; Table S2. Retrieval strategy; Table S3. Baseline characteristics of the included studies [46,47,48,49,50,51,52,53,54,55,56,57,58,59,60,61,62,63,64,65,66,67,68,69,70,71,72,73,74,75,76,77,78,79,80,81,82]; Table S4. Newcastle-Ottawa Quality Assessment Scale for the 42 cohort studies; Table S5. Seven risk factors are included in the systematic review and meta-analysis; Table S6. The clinical characteristics of pregnant women in the GDM and NGT groups in the 1st trimester; Table S7. Performance of the GDM risk prediction model at different cut-off values; Table S8. The comparison of the score-scaled GDM prediction model and logistic regression analysis of the GDM prediction model; Table S9. The comparison of the two ML models; Table S10. The validation of the published GDM models and models established in this study. Figure S1–S8: The forest plots of included variables in the score-scaled GDM prediction model; Figure S9: Funnel plot of the included variables in the score-scaled GDM prediction model; Figure S10: The ROC curves for the two ML models; Figure S11: The tree structure in the decision tree model; Figure S12: The tree structure in random forest model; Figure S13: Feature importance curve derived from the decision tree and random forest models.

## Abbreviations

GDM	gestational diabetes mellitus
ML	machine learning
AUC	area under the curve
NGT	normal glucose tolerance
OGTT	oral glucose tolerance test
pre-BMI	pre-pregnancy body mass index
GWG	gestational weight gain
TPO-Ab	thyroid peroxidase antibody
Tg-Ab	thyroglobulin antibody
ART	assisted reproductive technology
ALT	glutamic-pyruvic transaminase
AST	glutamic oxalacetic transaminase
CHO	total cholesterol
TG	triglyceride
HDL-C	high-density lipoprotein cholesterol
LDL-C	low-density lipoprotein cholesterol
