# Supplementary material for: Development and Validation of Risk Prediction Models for Gestational Diabetes Mellitus Using Four Different Methods
Source: Metabolites. 2022 Oct 29;12(11):1040. doi: 10.3390/metabo12111040 (PMC9697464; doi:10.3390/metabo12111040)
Supplement: Supplementary file 1 [file metabolites-12-01040-s001.zip › metabolites-1900040-supplementary.pdf]

Supplementary Table S1. Key words and derivative words

MeSH terms	Entry Terms
Diabetes Gestational	Diabetes, Pregnancy-Induced; Diabetes, Pregnancy Induced; Pregnancy-Induced Diabetes; Gestational Diabetes; Diabetes Mellitus, Gestational; Gestational Diabetes Mellitus
Risk factors	Factor, Risk; Risk Factor; Health Correlates; Correlates, Health; Risk Scores; Risk Score; Score, Risk; Risk Factor Scores; Risk Factor Score; Score, Risk Factor; Population at Risk; Populations at Risk
Cohort study	Cohort Study; Studies, Cohort; Study, Cohort; Concurrent Studies; Studies, Concurrent; Concurrent Study; Study, Concurrent; Closed Cohort Studies; Cohort Studies, Closed; Closed Cohort Study; Cohort Study, Closed; Study, Closed Cohort; Studies, Closed Cohort Analysis, Cohort; Cohort Analysis; Analyses, Cohort; Cohort Analyses; Historical Cohort Studies; Cohort Study, Historical; Historical Cohort Study; Study, Historical Cohort; Cohort Studies, Historical; Studies, Historical Cohort; Incidence Studies; Incidence Study; Studies, Incidence; Study, Incidence

Supplementary Table S2. Retrieval strategy

Website	Retrieval strategy		Number of papers
MEDLINE	(((((((((Diabetes, Gestational[MeSH Terms]) OR (Diabetes, Pregnancy-Induced[MeSH Terms])) OR (Diabetes, Pregnancy Induced[MeSH Terms])) OR (Pregnancy-Induced Diabetes[MeSH Terms])) OR (Gestational Diabetes[MeSH Terms])) OR (Diabetes Mellitus, Gestational[MeSH Terms])) OR (Gestational Diabetes Mellitus[MeSH Terms])) AND (((((((Diabetes, Gestational[MeSH Terms]) OR (Diabetes, Pregnancy-Induced[MeSH Terms])) OR (Diabetes, Pregnancy Induced[MeSH Terms])) OR (Pregnancy-Induced Diabetes[MeSH Terms])) OR (Gestational Diabetes[MeSH Terms])) OR (Diabetes Mellitus, Gestational[MeSH Terms])) OR (Gestational Diabetes Mellitus[MeSH Terms]))) AND (((((((((((((Risk Factors[MeSH Terms]) OR (Factor, Risk[MeSH Terms])) OR (Risk Factor[MeSH Terms])) OR (Health Correlates[MeSH Terms])) OR (Correlates, Health[MeSH Terms])) OR (Risk Scores[MeSH Terms])) OR (Risk Score[MeSH Terms])) OR (Score, Risk[MeSH Terms])) OR (Risk Factor Scores[MeSH Terms])) OR (Risk Factor Score[MeSH Terms])) OR (Score, Risk Factor[MeSH Terms])) OR (Population at Risk[MeSH Terms])) OR (Populations at Risk[MeSH Terms]))) AND ((((((((((((((((((((((((((((Cohort Studies[MeSH Terms]) OR (Cohort Study[MeSH Terms])) OR (Studies, Cohort[MeSH Terms])) OR (Study, Cohort[MeSH Terms])) OR (Concurrent Studies[MeSH Terms])) OR (Studies, Concurrent[MeSH Terms])) OR (Concurrent Study[MeSH Terms])) OR (Study, Concurrent[MeSH Terms])) OR (Closed Cohort Studies[MeSH Terms])) OR (Cohort Studies, Closed[MeSH Terms])) OR (Closed Cohort Study[MeSH Terms])) OR (Cohort Study, Closed[MeSH Terms])) OR (Study, Closed Cohort[MeSH Terms])) OR (Studies, Closed Cohort[MeSH Terms])) OR (Studies, Closed Cohort[MeSH Terms])) OR (Cohort Analysis[MeSH Terms])) OR (Analyses, Cohort[MeSH Terms])) OR (Cohort Analyses[MeSH Terms])) OR (Historical Cohort Studies[MeSH Terms])) OR (Cohort Study, Historical[MeSH Terms])) OR (Historical Cohort Study[MeSH Terms])) OR (Study, Historical Cohort[MeSH Terms])) OR (Cohort Studies, Historical[MeSH Terms])) OR (Studies, Historical Cohort[MeSH Terms])) OR (Incidence Studies[MeSH Terms])) OR (Incidence Study[MeSH Terms])) OR (Studies, Incidence[MeSH Terms])) OR (Study, Incidence[MeSH Terms]))		1650
Web of Science	1	TS=(Diabetes, Gestational OR Diabetes, Pregnancy-Induced OR Diabetes, Pregnancy Induced OR Pregnancy-Induced Diabetes OR Gestational Diabetes OR Diabetes Mellitus, Gestational OR G estational Diabetes Mellitus)<br><br>database = WOS, BCI, CSCD, DIIDW, INSPEC, KJD, MEDLINE, RSCI, SCIELO<br>time span = all year<br><br>retrieval language = automatic	40187
	2	TS=(Risk Factors OR Factor, Risk OR Risk Factor OR Health Correlates OR Correl ates, Health OR Risk Scores OR Risk Score OR Score, Risk OR Risk Factor Scores	2895742

		OR Risk Factor Score OR Score, Risk Factor OR Population at Risk OR Populations at Risk)<br>database = WOS, BCI, CSCD, DIIDW, INSPEC, KJD, MEDLINE, RSCI, SCIELO time span = all year<br>retrieval language = automatic	
	3	TS=(Cohort Studies OR Cohort Study OR Studies, Cohort OR Study, Cohort OR Concurrent Studies OR Studies, Concurrent OR Concurrent Study OR Study, Concurrent OR Closed Cohort Studies OR Cohort Studies, Closed OR Closed Cohort Study OR Cohort Study, Closed OR Study, Closed Cohort OR Studies, Closed Cohort OR Analysis, Cohort OR Cohort Analysis OR Analyses, Cohort OR Cohort Analyses OR Historical Cohort Studies OR Cohort Study, Historical OR Historical Cohort Study OR Study, Historical Cohort OR Cohort Studies, Historical OR Studies, Historical Cohort OR Incidence Studies OR Incidence Study OR Studies, Incidence OR Study, Incidence)<br>database = WOS, BCI, CSCD, DIIDW, INSPEC, KJD, MEDLINE, RSCI, SCIELO time span = all year<br>retrieval language = automatic	1929117
	4	#3 AND #2 AND #1<br>database = WOS, BCI, CSCD, DIIDW, INSPEC, KJD, MEDLINE, RSCI, SCIELO time span = all year<br>retrieval language = automatic	5085
Embase	('diabetes, gestational'/exp OR 'diabetes, gestational' OR 'diabetes, pregnancy-induced' OR 'diabetes, pregnancy induced' OR 'pregnancy-induced diabetes' OR 'gestational diabetes'/exp OR 'gestational diabetes' OR 'diabetes mellitus, gestational' OR 'gestational diabetes mellitus'/exp OR 'gestational diabetes mellitus') AND ('risk factors'/exp OR 'risk factors' OR 'factor, risk' OR 'risk factor'/exp OR 'risk factor' OR 'health correlates' OR 'correlates, health' OR 'risk scores' OR 'risk score'/exp OR 'risk score' OR 'score, risk' OR 'risk factor scores' OR 'risk factor score' OR 'score, risk factor' OR 'population at risk'/exp OR 'population at risk' OR 'populations at risk') AND ('cohort studies'/exp OR 'cohort studies' OR 'cohort study'/exp OR 'cohort study' OR 'studies, cohort' OR 'study, cohort' OR 'concurrent studies' OR 'studies, concurrent' OR 'concurrent study' OR 'study, concurrent' OR 'closed cohort studies' OR 'cohort studies, closed' OR 'closed cohort study' OR 'cohort study, closed' OR 'study, closed cohort' OR 'studies, closed cohort' OR 'analysis, cohort'/exp OR 'analysis, cohort' OR 'cohort analysis'/exp OR 'cohort analysis' OR 'analyses, cohort' OR 'cohort analyses' OR 'historical cohort studies' OR 'cohort study, historical' OR 'historical cohort study' OR 'study, historical cohort' OR 'cohort studies, historical' OR 'studies, historical cohort' OR 'incidence studies' OR 'incidence study' OR 'studies, incidence' OR 'study, incidence')		2202

Total	8937

Supplementary Table S3. Baseline characteristics of the included studies

First Author/Year/Country or region(continent)	Source of cohort	Study design and period	Sample size	Risk factors for GDM development
Qi 2020[1]	Cohort of pregnant women (Sichuan, China)	Retrospective 2013 - 2014	8356	GWG at the 1st trimester
Hedderson 2010[2]	Cohort of kaiser permanente(Northern California, USA)	Retrospective 1996-1998	1145	GWG at the 1st trimester
Zhong 2017[3]	Cohort of mother-infant pairs[Wuhan, China(TMCHC study)]	Prospective 2013 - 2016	2090	GWG at the 1st trimester
Chen 2015[4]	Nurses' Health Study II (USA)	Prospective, 1989 - 2001	27482	Age at menarche
Dishi 2011[5]	Omega study (Sweden)	Prospective, 1996 - 2008	3490	Age at menarche
Li 2016[6]	HBC Study (Wuhan, China)	Prospective, 2012 - 2014	6900	Age at menarche
Shen 2016[7]	NHANES Study (USA.)	Retrospective, 2007-2012	5914	Age at menarche
Hao 2016[8]	Cohort of pregnant women (Shanghai, China)	Prospective, 2012 - 2014	7084	Thyroid Diseases
Li 2020[9]	Women and Children's Health Care Center System (Qingdao, China)	Retrospective, 2018 - 2019	17145	Thyroid Diseases, gestational age, pre-BMI
Mannisto 2013[10]	Consortium on Safe Labor (USA)	Retrospective, 2002 - 2008	223512	Thyroid Diseases
Yang 2016[11]	Cohort of pregnant women (Shanghai, China)	Retrospective, 2013 - 2015	27513	Thyroid Diseases
Lei 2019[12]	Cohort of pregnant women (Beijing, China)	Retrospective, 2013 - 2015	9024	ART
Barua 2016[13]	Cohort of pregnant women (western Sydney, Australia)	Retrospective, 2007 - 2010	50375	ART
Hu 2019[14]	Cohort of pregnant women (Shanghai, China)	Retrospective, 2014 - 2017	1022	ART
Nagata 2019[15]	Japan environment and children's study (Japan)	Retrospective, 2011 - 2014	97592	ART
Shevell 2005[16]	The first and second trimester evaluation of risk trial (a international multicenter investigation)	Prospective, 1999 - 2002	36062	ART

Silberstein 2013[17]	Cohort of pregnant women (Israel)	Retrospective, 1998 - 2006	174781	ART
Stern 2014[18]	Cohort of pregnant women (Massachusetts, USA)	Retrospective, 2004 - 2008	305774	ART
Wang 2013[19]	Cohort of pregnant women (Australia)	Retrospective, 2007 - 2009	400392	ART
Cosson 2013[20]	Cohort of pregnant women (an international multicenter investigation)	Prospective, 2002 - 2010	17344	T2DM family history
Schoenaker 2018[21]	Australian Longitudinal Study on Women's Health (Australia)	Prospective, 1996 - 2015	6504	T2DM family history
Hossein 2018[22]	Cohort of pregnant women (Iran)	Prospective, 2015 - 2017	929	T2DM family history
Larrabure-Torrealva 2018[23]	Cohort of pregnant women (Peru)	Retrospective, 2013 - 2014	1300	T2DM family history
Leng 2014[24]	Cohort of pregnant women (Tianjin, China)	Retrospective, 2010 - 2012	17808	T2DM family history
Pirjani 2016[25]	Cohort of pregnant women (Iran)	Prospective, 2012 - 2013	256	T2DM family history, pre-BMI
Schaefer 2018[26]	Cohort of pregnant women (Guangzhou, China)	Prospective, 2012 - 2015	8381	T2DM family history
Shahbazian 2016[27]	Cohort of pregnant women (Iran)	Prospective, 2014 - 2015	750	T2DM family history
Wang 2019[28]	Cohort of pregnant women (Sichuan, China)	Retrospective, 2016 - 2018	3608	T2DM family history, gestational age
Yan 2016[29]	Cohort of pregnant women (Xiamen, China)	Retrospective, 2011 - 2018	78572	T2DM family history
Khaili 2013[30]	Cohort of pregnant women (UK)	Retrospective	76161	Maternal age
Londero 2019[31]	Cohort of pregnant women (Italy)	Retrospective, 2001 - 2014	22933	Gestational age
Sweeting 2017[32]	Cohort of pregnant women (Sydney, Australia)	Retrospective, 2011 - 2013	980	Gestational age, pre-BMI
Wang 2017[33]	Cohort of pregnant women (Beijing, China)	Retrospective, 2013	14970	Gestational age
Koo 2011[34]	Cohort of pregnant women (Korea)	Retrospective, 2005 - 2008	29760	Gestational age
Sun 2020[35]	CPWCS-PUMC cohort study (Beijing, China)	Retrospective, 2017 - 2018	3172	pre-BMI
Rodríguez-Mesa 2019[36]	Cohort of pregnant women (Spain)	Retrospective, 2014 - 2015	710	pre-BMI
Shaukat 2019[37]	PHCC Database (an international multicenter investigation)	Retrospective, 2016 - 2017	1134	pre-BMI

Gao 2017[38]	Cohort of pregnant women (Changsha, China)	Retrospective, 2015	919	pre-BMI
Laine 2017[39]	Cohort of pregnant women (Vantaa, Finland)	Retrospective, 2009 - 2015	7750	pre-BMI
Yong 2020[40]	Cohort of pregnant women (Malaysia)	Retrospective, 2010 - 2012	1951	pre-BMI
Hashemi-Nazari 2018[41]	Cohort of pregnant women (Iran)	Retrospective, 2015 - 2016	1009	pre-BMI
Shao 2020[42]	Cohort of pregnant women (Zhoushan, China)	Retrospective, 2011-2018	3318	pre-BMI

Supplementary Table S4. Newcastle-Ottawa Quality Assessment Scale for the 42 cohort studies

**Study (First author/year)**	**Selection**				**Comparability**	**Outcome**			**Total scores**
	Representativeness of the exposed cohort	Selectivity of the non-exposed cohort	Ascertainment of exposure	Demonstration that outcome of interest was not present at start of study		Assessment of outcome	Was the follow-up long enough for outcomes to occur	Adequacy of follow-up of cohorts	
Qi 2020	★	★	★	★	★★	★	★	★	9
Hedderson 2010	★	★	★	★	★★	★	★		8
Zhong 2017	★	★	★	★	★★	★	★		8
Chen 2015	★	★	★	★	★	★	★		7
Dishi 2011	★	★	★	★	★	★	★		7
Li 2016	★	★	★	★	★	★	★		7
Shen 2016	★	★	★	★	★★	★	★		8
Hao 2016	★	★	★	★	★★	★	★	★	9
Li 2020	★	★	★	★	★★	★	★		8
Mannisto 2013	★	★	★	★	★★	★	★		8
Yang 2016	★	★	★	★	★★	★	★	★	9
Lei 2019	★	★	★	★	★	★	★		7
Silberstein 2013	★	★	★	★	★	★	★		7
Stern 2014	★	★	★	★	★	★	★	★	8
Wang 2013	★	★	★	★	★★	★	★	★	9

Barua 2016	★	★	★	★	★	★	★	★	8
Hu 2019	★	★	★	★	★	★	★		7
Nagata 2019	★	★	★	★	★★	★	★	★	9
Shevell 2005	★	★	★	★	★★	★	★	★	9
Khaili 2013	★	★	★	★	★	★	★	★	8
Londero 2019	★	★	★	★	★★	★	★	★	9
Sweeting 2017	★	★	★	★	★★	★	★	★	9
Wang 2017	★	★	★	★	★★	★	★	★	9
Koo 2011	★	★	★	★	★	★	★	★	8
Wang 2019	★	★	★	★	★★	★	★	★	9
Leng 2014	★	★	★	★	★★	★	★	★	9
Schoenaker 2018	★	★	★	★	★★	★	★	★	9
Schaefer 2018	★	★	★	★	★	★	★	★	8
Shahbazian 2016	★	★	★	★	★	★	★		7
Pirjani 2016	★	★	★	★	★	★	★	★	8
Cosson 2013	★	★	★	★	★★	★	★	★	9
Hossein 2018	★	★	★	★	★★	★	★	★	9

Larrabure-Torrealva2018	★	★	★	★	★★	★	★	★	9
Yan 2016	★	★	★	★	★	★	★	★	8
Sun 2020	★	★	★	★	★★	★	★		8
Rodríguez-Mesa 2019	★	★	★	★	★	★	★		7
Gao 2017	★	★	★	★	★	★	★	★	8
Laine 2017	★	★	★	★	★	★	★		7
Shaukat 2019	★	★	★	★	★★	★	★		8
Yong 2020	★	★	★	★	★★	★	★		8
Hashemi-Nazari 2018	★	★	★	★	★	★	★		7
Shao 2020	★	★	★	★	★★	★	★		8

Supplementary Table S5. Seven risk factors included in the systematic review and meta-analysis

Risk factors	First author/Year	Sample size	Definition of risk factor	OR	95%CI	*P* value
GWG at the 1st trimester	Qi 2020	632	Above IOM recommended GWG at the 1st trimester	1.07	0.94 - 1.21	＞0.05
GWG at the 1st trimester	Hedderson 2010	245	Above IOM recommended GWG at the 1st trimester	1.53	1.09 - 2.15	＜0.05
GWG at the 1st trimester	Zhong 2017	921	Above IOM recommended GWG at the 1st trimester	0.59	0.33 - 1.15	＞0.05
Thyroid relative antibodies (+)	Hao 2016	281	TSH(H)TgAb(+)	1.44	1.14 - 1.84	＜0.05
Thyroid relative antibodies (+)	Hao 2016	648	TSH(N)TPOAb(+)	1.65	1.43 - 1.92	＜0.05
Thyroid relative antibodies (+)	Hao 2016	78	TSH(H)TPOAb(+)	3.38	2.63 - 3.45	＜0.05
Thyroid relative antibodies (+)	Li 2020	578	TSH 0.1 - 2.5 mIU/L at the 1st trimester or 0.2 - 3.0 mIU/L, TgAb/TPOAb(+) at the 2nd or 3rd trimester	1.47	1.11 - 1.96	< 0.05
Thyroid relative antibodies (+)	Mannisto 2013	3183	TgAb(+)	1.57	1.33 - 1.86	< 0.05
Thyroid relative antibodies (+)	Yang 2016	393	TPOAb(+)	1.10	0.98 - 1.23	0.118
Age at menarche	Chen 2015	8730	Age at menarche ≤11 yr	1.34	1.14 - 1.58	< 0.05
Age at menarche	Dishi 2011	505	Age at menarche ≤11 yr	0.82	0.51 - 1.32	> 0.05
Age at menarche	Li 2016	379	Age at menarche ≤11 yr	1.41	1.06 - 1.87	< 0.05
Age at menarche	Shen 2016	115	Age at menarche	1.75	1.10-2.79	< 0.05

			≤11 yr			
ART	Lei 2019	2256	IVF+ICSI	1.85	1.24 - 2.78	< 0.01
ART	Barua 2016	1721	IVF ICSI IUI OI	1.509	1.30 - 1.75	< 0.001
ART	Hu 2019	237	ICSI	0.859	0.59 - 1.25	0.859
ART	Hu 2019	353	Secondary infertility	1.187	0.85 - 1.65	0.311
ART	Hu 2019	358	Fresh cycles	1.382	1.01 - 1.90	0.046
ART	Nagata 2019	3939	ovulation induction	0.97	0.8 - 1.19	> 0.05
ART	Nagata 2019	1476	IVT-ET	0.99	0.75 - 1.31	> 0.05
ART	Nagata 2019	1671	ICSI	0.97	0.74 - 1.27	> 0.05
ART	Shevell 2005	1222	Ovulation Induction	1.5	1.1 - 2.20	0.01
ART	Shevell 2005	554	IVF	0.5	0.2 - 1.0o	0.06
ART	Silberstein 2013	1294	IVF	1.77	1.52 - 2.07	< 0.001
ART	Stern 2014	1901	male factor	1.15	0.96 - 1.38	> 0 .05
ART	Stern 2014	406	endometriosis	0.93	0.62 - 1.39	> 0.05
ART	Stern 2014	676	ovulation disorders	2.17	1.72 - 2.73	< 0.05
ART	Stern 2014	706	tubal	1.42	1.09 - 1.84	< 0.05
ART	Wang 2013	13732	IVF, ICSI or gamete intrafallopian transfer	1.26	1.18 - 1.36	< 0.05
T2DM family history	Cosson 2013	3588	T2DM family history	1.14	1.03 - 1.27	< 0.05
T2DM family history	Schoenaker 2018	544	T2DM family history	1.53	0.79 - 2.95	> 0.05
T2DM family history	Hossein 2018	56	Early onset	3.10	1.90 - 5.20	< 0.05
T2DM family history	Hossein 2018	34	Late onset	2.20	1.20 - 3.90	< 0.05
T2DM family history	Larrabure-Torrealva 2018	466	T2DM family history	1.51	1.1 - 2.07	< 0.05
T2DM family history	Leng 2014	191	T2DM family history	1.61	1.36 - 1.91	< 0.05
T2DM family history	Pirjani 2016	91	T2DM family history	2.01	1.13 - 3.56	0.016
T2DM family history	Schaefer 2018	842	T2DM family history	1.51	1.25 - 1.82	< 0.05
T2DM family history	Shahbazian 2016	87	T2DM family history	2.47	1.33 - 4.59	< 0.05
T2DM family history	Wang 2019	299	T2DM family history	2.52	1.94 - 3.27	< 0.05

T2DM family history	Yan 2016	1825	T2DM family history	1.97	1.78 - 2.19	< 0.001
Maternal age	Khaili 2013	35 - 39	16325	1.62	1.43 - 1.83	< 0.05
Maternal age	Khaili 2013	≥ 40	4061	1.88	1.55 - 2.29	< 0.05
Maternal age	Londero 2019	≥ 40	1552	1.71	1.37 - 2.12	< 0.05
Maternal age	Sweeting 2017	322	30 - 34	1.18	0.80 - 1.76	> 0.05
Maternal age	Sweeting 2017	404	≥ 35	1.49	1.03 - 2.16	< 0.05
Maternal age	Wang 2017	4365	30-34	1.51	1.37 - 1.66	< 0.05
Maternal age	Wang 2017	1017	35-40	2.09	1.79 - 2.46	< 0.05
Maternal age	Wang 2017	155	≥40	2.17	1.49 - 3.17	< 0.05
Maternal age	Li 2020	5893	30-40	1.24	1.05 - 1.49	< 0.05
Maternal age	Li 2020	3207	≥35	2.18	1.84 - 2.59	< 0.05
Maternal age	Koo 2011	15496	30-34	2.00	1.60 - 2.60	< 0.001
Maternal age	Koo 2011	5665	35 - 39	3.60	2.80 - 4.80	< 0.001
Maternal age	Koo 2011	649	> 40	5.10	3.30 - 7.80	< 0.001
Maternal age	Wang 2019	1606	30 - 34	1.88	1.54 - 2.30	< 0.05
Maternal age	Wang 2019	527	≥35	2.88	2.24 - 3.71	< 0.05
pre-BMI	Sun 2020	401	overweight	2.01	1.47 - 2.71	< 0.001
pre-BMI	Sun 2020	59	obese	1.91	0.94 - 3.91	0.075
pre-BMI	Rodríguez-Mesa 2019	109	obese	3.61	2.10 - 6.20	< 0.001
pre-BMI	Shaukat 2019	240	overweight (non-Arabs)	1.23	0.76 - 2.00	> 0.05
pre-BMI	Shaukat 2019	159	overweight (Arabs)	1.24	0.84 - 1.82	> 0.05
pre-BMI	Shaukat 2019	114	obese (Arabs)	2.38	1.51 - 3.84	< 0.05
pre-BMI	Shaukat 2019	116	obese(non-Arabs)	1.60	0.97 - 2.65	> 0.05
pre-BMI	Gao 2017	141	overweight and obese	2.19	1.02 - 4.76	< 0.05
pre-BMI	Laine 2017	758	obese	5.36	4.53 - 6.36	< 0.05
pre-BMI	Yong 2020	848	overweight and obese	1.44	1.04 - 1.81	< 0.05
pre-BMI	Hashemi-Nazari 2018	Not given	overweight	1.53	0.96 - 2.43	0.05
pre-BMI	Hashemi-Nazari 2018	Not given	obese	2.19	1.30 - 3.68	0.003
pre-BMI	Shao 2020	342	overweight	1.85	1.41 - 2.43	< 0.001
pre-BMI	Shao 2020	70	obese	1.91	1.12 - 3.27	< 0.05
pre-BMI	Li 2020	2980	overweight	1.63	1.26 - 2.11	< 0.05
pre-BMI	Li 2020	611	obese	1.3	0.75 - 2.26	< 0.05
pre-BMI	Sweeting 2017	255	obese	1.2	0.85 - 1.70	> 0.05
pre-BMI	Pirjani 2016	Not given	overweight	1.01	0.54 - 1.92	> 0.05
pre-BMI	Pirjani 2016	40	obese	2.74	1.28 - 5.8	< 0.05

Supplementary Table S6. The clinical characteristics of pregnant women in the GDM and NGT groups at the 1st trimester

**Variables**	**NGT**	**GDM**	***P* value**
Number	723	352	-
Maternal age	30.83 ± 0.41	31.13 ± 0.57	0.017
T2DM family history	46 (6.4)	57 (16.2)	＜ 0.001
pre-BMI	21.02 ± 0.28	22.89 ± 0.40	0.009
Age at menarche≤11yr	36 (5.0)	48 (13.6)	＜ 0.001
ART	22 (3.0)	62 (17.6)	＜ 0.001
Thyroid antibodies (+) [TPOAb/TgAb]	45 (6.2)	123 (34.9)	＜ 0.001
Above IOM recommended GWG at the 1st trimester	73 (10.1)	118 (33.5)	＜ 0.001
History of macrosomia	15 (2.1)	24 (6.8)	＜ 0.001
Parity	1.47 ± 0.05	1.51 ± 0.08	0.841
FBG (mmol/L)	4.93 ± 0.33	5.06 ± 0.34	＜ 0.05
Vitamin B12（pg/mL）	64.51 ± 16.01	64.60 ± 18.92	0.171
Ferritin（ng/mL）	46.58 ± 4.17	48.11 ± 3.54	0.210
Total protein（g/L）	69.85 ± 0.50	69.44 ± 0.50	0.793
Albumin（g/L）	40.71 ± 0.25	40.05 ± 0.39	0.125
Globulin（g/L）	29.15 ± 0.42	28.39 ± 0.37	0.060
ALT(U/L）	17.53 ± 1.38	17.95 ± 2.01	0.105
AST(U/L）	18.92 ± 0.82	18.25 ± 1.10	0.121
CHO（mmol/L）	4.16 (3.12 – 5.35)	4.21(3.38 – 5.72)	0.715
TG（mmol/L）	1.47 ± 0.07	1.51 ± 0.88	0.977
HDL-C（mmol/L）	1.65 ± 0.04	1.61 ± 0.05	0.268
LDL-C（mmol/L）	2.32 ± 0.06	2.36 ± 0.08	0.900

Note. Data were presented as n (%), mean (SD, standard deviation). * One-way ANOVA was used for continuous variables showing normal distribution, and the Chi-square test (or Fisher's Exact test) was used for categorical variables. GDM, gestational diabetes mellitus; NGT, normal glucose tolerance; pre-BMI, pre-pregnancy body mass index; ART, assisted reproductive technology; TPOAb, thyroid peroxidase antibody; TGAb, anti-thyroglobulin antibody; FBG: fasting blood glucose; ALT, alanine aminotransferase; AST, aspartate aminotransferase: CHO, total cholesterol; TG, triglyceride; HDL-C, high density lipoprotein cholesterol; LDL-C: low density lipoprotein cholesterol.

Supplementary Table S7. Performance of the GDM risk prediction model at different cut-off values

Cut-off value	Sensitivity	Specificity	Youden index
1	0.949	0.314	0.263
2.5	0.903	0.336	0.239
3.5	0.903	0.342	0.245
4.5	0.855	0.375	0.23
5.5	0.736	0.674	0.41
6.5	0.733	0.696	0.429
7.5	0.653	0.737	0.39
8.5	0.628	0.817	0.445
9.5	0.585	0.881	0.466
10.5	0.474	0.899	0.373
11.5	0.418	0.923	0.341
12.5	0.324	0.943	0.267
13.5	0.264	0.957	0.221
14.5	0.23	0.97	0.2
15.5	0.173	0.978	0.151
16.5	0.145	0.986	0.131
17.5	0.114	0.989	0.103
18.5	0.08	0.993	0.073
19.5	0.057	0.994	0.051
20.5	0.045	0.997	0.042
21.5	0.034	0.999	0.033
22.5	0.028	1	0.028
23.5	0.014	1	0.014
25	0.011	1	0.011
26.5	0.009	1	0.009
27.5	0.003	1	0.003
29	0	1	0

Supplementary Table S8. The comparison of the score-scaled GDM prediction model and logistic regression analysis of the GDM prediction model

**Variables**	**NRI**	***P***	**IDI**	***P***
model 1 - model 2	0.208	< 0.001	0.045	< 0.001

Model 1, the score scaled GDM risk prediction model; model 2, the logistic regression analysis of GDM risk prediction model. The test was conducted by Z test. A *P* value < 0.05 is considered a significant.

Supplementary Table S9. The comparison of the two ML models.

Models	AUC (training set)	AUC (validation set)	Precision	Recall	Fi-score	Accuracy	Specificity
Decision Tree	0.825	0.816	0.799	0.709	0.751	76.8%	0.825
Random Forest	0.823	0.827	0.768	0.724	0.745	75.5%	0.785

ML models, machine learning models. *P* value < 0.05 was considered statistically significant.

Supplementary Table S10. The validation of the published GDM models and models established by in this study

Author/Year	Variables	AUC (95%CI)	*P*	Sensitivity	Specificity
Published models					
Zheng 2019	Age, BMI, FPG, TG	0.354 (0.266-0.442)	0.092	0.923	0.023
Guo 2020	Age, BMI, FPG, diabetes family history	0.716 (0.625-0.806)	＜0.001	0.102	1.0
Zhang 2020	Age, BMI, HbA1c, TG	0.709 (0.614-0.804)	＜0.001	0.102	1.0
Models established in this study					
Score-scared model	-	0.769(0.681-0.858)	＜0.001	53.8	87.8
Logistic regression model	-	0.841(0.736-0.891)	＜0.001	28.2	99.4
Decision tree model	-	0.777(0.726-0.829)	＜0.001	63.2	80.6
Random forest model	-	0.740(0.684-0.795)	＜0.001	65.1	74.8

Supplementary Figure S1. Age

Study or Subgroup	log[Risk Ratio]	SE	Weight	Odds Ratio IV, Random, 95% CI
**30-34 yr vs.<30 yr**				
Koo 2011	0.69314718	0.12385404	23.4%	2.00 [1.57, 2.55]
Sweeting 2017	0.16551444	0.20113708	13.9%	1.18 [0.80, 1.75]
Wang 2017	0.41409444	0.049158	35.8%	1.51 [1.37, 1.67]
Wang 2019	0.63127178	0.10232824	26.9%	1.88 [1.54, 2.30]
**Subtotal (95% CI)**			**100.0%**	**1.65 [1.38, 1.98]**
Heterogeneity: Tau² = 0.02; Chi² = 9.31, df = 3 (P = 0.03); I² = 68%				
Test for overall effect: Z = 5.40 (P < 0.00001)				
**35-39 yr vs.<30 yr**				
Khaili 2013	0.48242615	0.06291876	18.8%	1.62 [1.43, 1.83]
Koo 2011	1.28093384	0.13749911	15.7%	3.60 [2.75, 4.71]
Li 2020	0.77932488	0.08721742	18.0%	2.18 [1.84, 2.59]
Sweeting 2017	0.39877612	0.18891567	13.2%	1.49 [1.03, 2.16]
Wang 2017	0.73907611	0.08116028	18.2%	2.09 [1.79, 2.46]
Wang 2019	1.05779029	0.12871327	16.1%	2.88 [2.24, 3.71]
**Subtotal (95% CI)**			**100.0%**	**2.20 [1.75, 2.78]**
Heterogeneity: Tau² = 0.07; Chi² = 41.10, df = 5 (P < 0.00001); I² = 88%				
Test for overall effect: Z = 6.67 (P < 0.00001)				
**≥40 yr vs.<30 yr**				
Khaili 2013	0.63127178	0.09956553	28.0%	1.88 [1.55, 2.29]
Koo 2011	1.62924054	0.2194391	21.5%	5.10 [3.32, 7.84]
Londero 2019	0.53649337	0.11137892	27.4%	1.71 [1.37, 2.13]
Wang 2017	0.77656879	0.19200696	23.1%	2.17 [1.49, 3.17]
**Subtotal (95% CI)**			**100.0%**	**2.35 [1.62, 3.40]**
Heterogeneity: Tau² = 0.12; Chi² = 20.72, df = 3 (P = 0.0001); I² = 86%				
Test for overall effect: Z = 4.53 (P < 0.00001)				

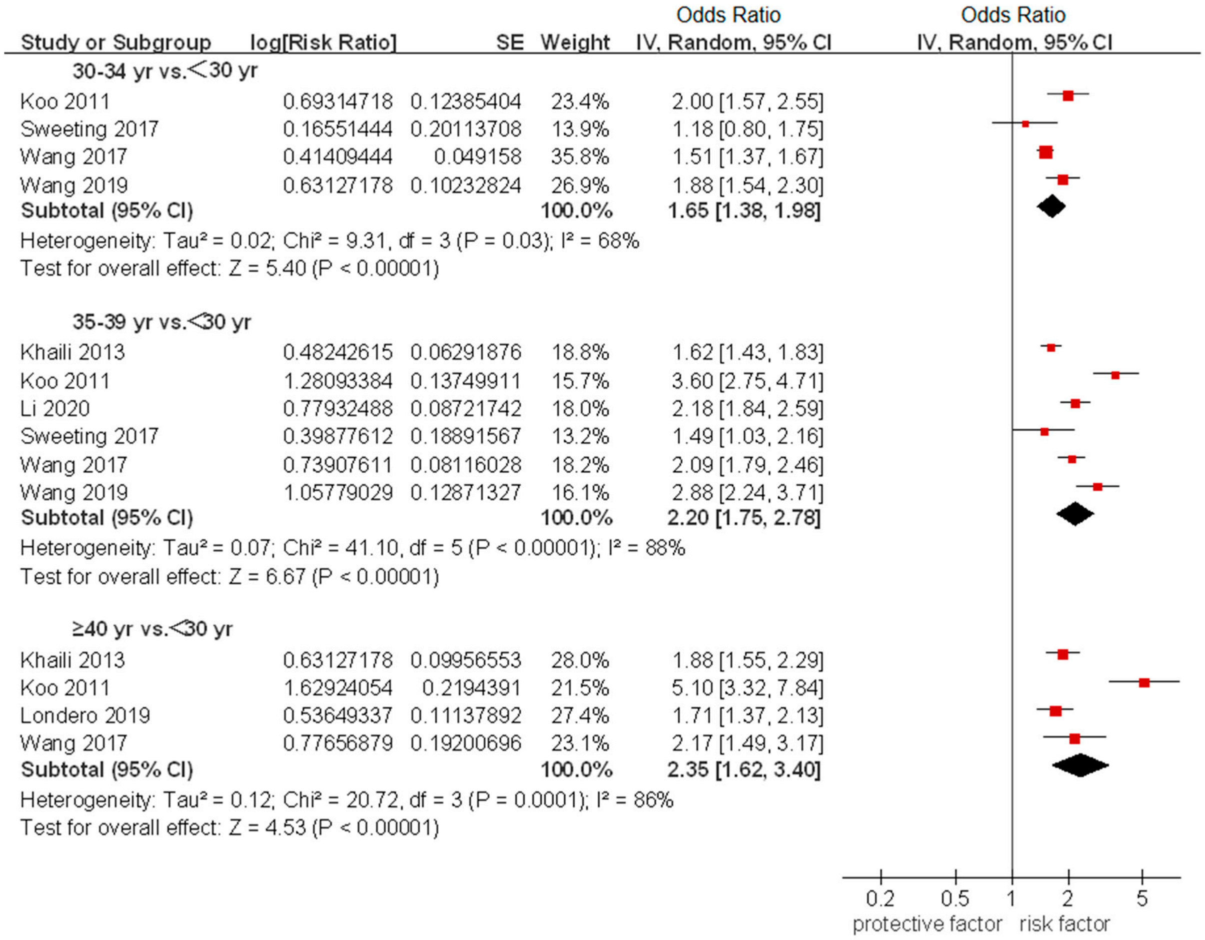

Supplementary Figure S2. T2DM family history

Study or Subgroup	log[Risk Ratio]	SE	Weight	Odds Ratio IV, Random, 95% CI
**T2DM family history vs. no T2DM family history**				
Cosson 2013	0.13102826	0.05343319	12.6%	1.14 [1.03, 1.27]
Hossein 2018 (early onset)	1.13140211	0.25683794	7.2%	3.10 [1.87, 5.13]
Hossein 2018 (late onset)	0.78845736	0.3006773	6.1%	2.20 [1.22, 3.97]
Larabure-Torreal 2018	0.41210965	0.16128531	9.8%	1.51 [1.10, 2.07]
Leng 2014	0.47623418	0.08663738	11.9%	1.61 [1.36, 1.91]
Pirjani 2016	0.69813472	0.29274054	6.3%	2.01 [1.13, 3.57]
Schaefer 2018	0.41210965	0.09584004	11.7%	1.51 [1.25, 1.82]
Schoenaker 2018	0.42526774	0.33610396	5.4%	1.53 [0.79, 2.96]
Shahbazian 2016	0.90421815	0.31599517	5.8%	2.47 [1.33, 4.59]
Wang 2019	0.92346494	0.13287705	10.7%	2.52 [1.94, 3.27]
Yan 2016	0.67803354	0.05287964	12.6%	1.97 [1.78, 2.19]
**Subtotal (95% CI)**			**100.0%**	**1.81 [1.48, 2.21]**
Heterogeneity: Tau² = 0.08; Chi² = 78.10, df = 10 (P < 0.00001); I² = 87%				
Test for overall effect: Z = 5.80 (P < 0.00001)				
**Sensitivity analysis**				
Hossein 2018 (late onset)	0.78845736	0.3006773	5.2%	2.20 [1.22, 3.97]
Larabure-Torreal 2018	0.41210965	0.16128531	14.4%	1.51 [1.10, 2.07]
Pirjani 2016	0.69813472	0.29274054	5.5%	2.01 [1.13, 3.57]
Schaefer 2018	0.41210965	0.09584004	26.6%	1.51 [1.25, 1.82]
Schoenaker 2018	0.42526774	0.33610396	4.3%	1.53 [0.79, 2.96]
Shahbazian 2016	0.90421815	0.31599517	4.8%	2.47 [1.33, 4.59]
Yan 2016	0.67803354	0.05287964	39.3%	1.97 [1.78, 2.19]
**Subtotal (95% CI)**			**100.0%**	**1.78 [1.54, 2.05]**
Heterogeneity: Tau² = 0.01; Chi² = 9.04, df = 6 (P = 0.17); I² = 34%				
Test for overall effect: Z = 7.94 (P < 0.00001)				

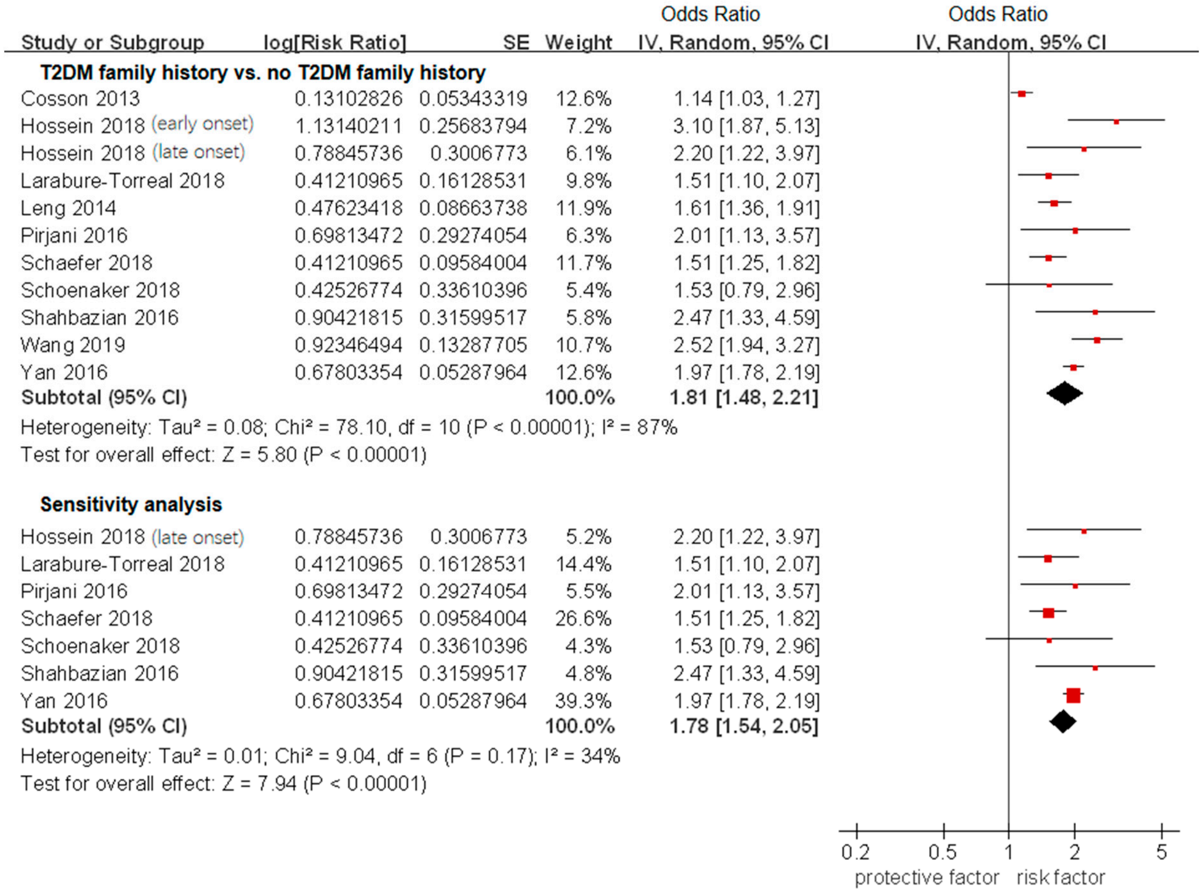

Supplementary Figure S3. Pre-BMI overweight

Study or Subgroup	log[Risk Ratio]	SE	Weight	Odds Ratio IV, Fixed, 95% CI
**pre-BMI overweight vs. normal weight**				
Gao2017	0.78390154	0.39297067	2.1%	2.19 [1.01, 4.73]
Hashemi-Nazari 2018	0.42526774	0.23691665	5.8%	1.53 [0.96, 2.43]
Li 2020	0.48858002	0.13152455	18.7%	1.63 [1.26, 2.11]
Pirjani12016	0.00995033	0.32359983	3.1%	1.01 [0.54, 1.90]
Shao 2020	0.61518564	0.13885244	16.8%	1.85 [1.41, 2.43]
Shaukat 2019(non-Arabs)	0.20701417	0.24683266	5.3%	1.23 [0.76, 2.00]
Shaukat 2019(Arabs)	0.21511138	0.19724232	8.3%	1.24 [0.84, 1.83]
Sun 2020	0.69813472	0.15604241	13.3%	2.01 [1.48, 2.73]
Sweeting 2017	0.18232156	0.17682326	10.4%	1.20 [0.85, 1.70]
Yong 2020	0.36464311	0.1413536	16.2%	1.44 [1.09, 1.90]
**Subtotal (95% CI)**			**100.0%**	**1.55 [1.38, 1.73]**

Heterogeneity: Chi² = 11.59, df = 9 (P = 0.24); I² = 22%
Test for overall effect: Z = 7.66 (P < 0.00001)

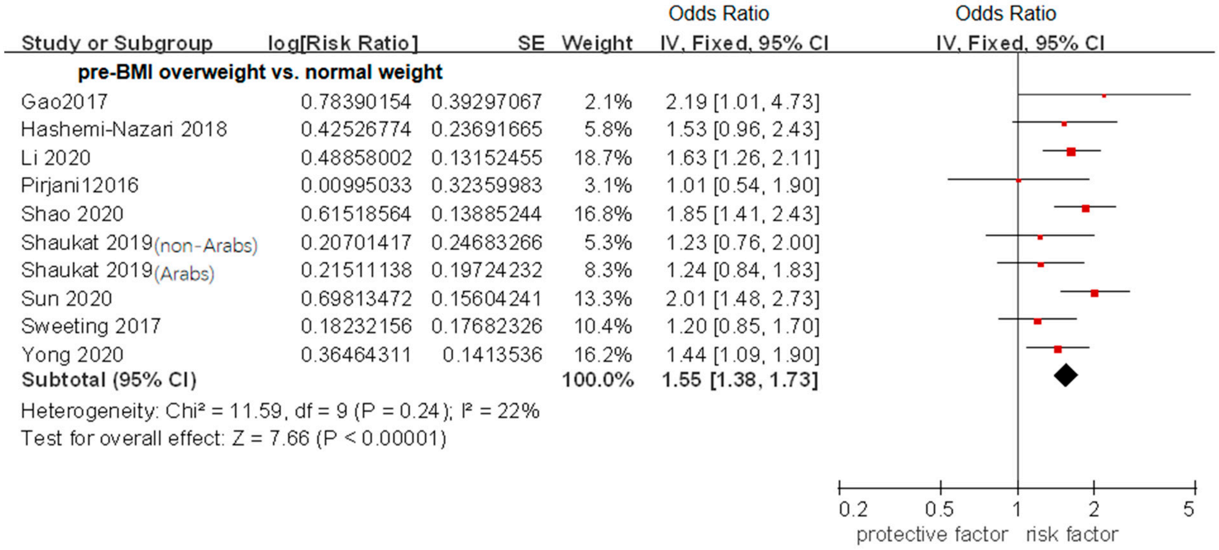

Supplementary Figure S4. Pre-BMI obesity and sensitivity analysis

Study or Subgroup	log[Risk Ratio]	SE	Weight	Odds Ratio IV, Fixed, 95% CI
**pre-BMI obese vs. normal weight**				
Hashemi-Nazari 2018	0.78390154	0.26544604	5.0%	2.19 [1.30, 3.68]
Laine12017	1.67896398	0.08655776	47.0%	5.36 [4.52, 6.35]
Li 2020	0.26236426	0.28138951	4.4%	1.30 [0.75, 2.26]
Pirjani12016	1.00795792	0.38895324	2.3%	2.74 [1.28, 5.87]
Rodríguez-Mesa 2019	1.28370777	0.27617652	4.6%	3.61 [2.10, 6.20]
Shao 2020	0.64710324	0.27333196	4.7%	1.91 [1.12, 3.26]
Shaukat 2019 (Arabs)	0.86710049	0.23810273	6.2%	2.38 [1.49, 3.80]
Shaukat 2019 (non-Arabs)	0.47000363	0.25638236	5.4%	1.60 [0.97, 2.64]
Sun 2020	0.64710324	0.36362571	2.7%	1.91 [0.94, 3.90]
Yong 2020	0.36464311	0.1413536	17.6%	1.44 [1.09, 1.90]
**Subtotal (95% CI)**			**100.0%**	**3.05 [2.71, 3.42]**
Heterogeneity: Chi² = 93.82, df = 9 (P < 0.00001); I² = 90%				
Test for overall effect: Z = 18.77 (P < 0.00001)				
**Sensitivity analysis**				
Hashemi-Nazari 2018	0.78390154	0.26544604	10.3%	2.19 [1.30, 3.68]
Li 2020	0.26236426	0.28138951	9.2%	1.30 [0.75, 2.26]
Pirjani12016	1.00795792	0.38895324	4.8%	2.74 [1.28, 5.87]
Shao 2020	0.64710324	0.27333196	9.8%	1.91 [1.12, 3.26]
Shaukat 2019 (non-Arabs)	0.47000363	0.25638236	11.1%	1.60 [0.97, 2.64]
Shaukat 2019 (Arabs)	0.86710049	0.23810273	12.8%	2.38 [1.49, 3.80]
Sun 2020	0.64710324	0.36362571	5.5%	1.91 [0.94, 3.90]
Yong 2020	0.36464311	0.1413536	36.5%	1.44 [1.09, 1.90]
**Subtotal (95% CI)**			**100.0%**	**1.73 [1.46, 2.05]**
Heterogeneity: Chi² = 7.00, df = 7 (P = 0.43); I² = 0%				
Test for overall effect: Z = 6.43 (P < 0.00001)				

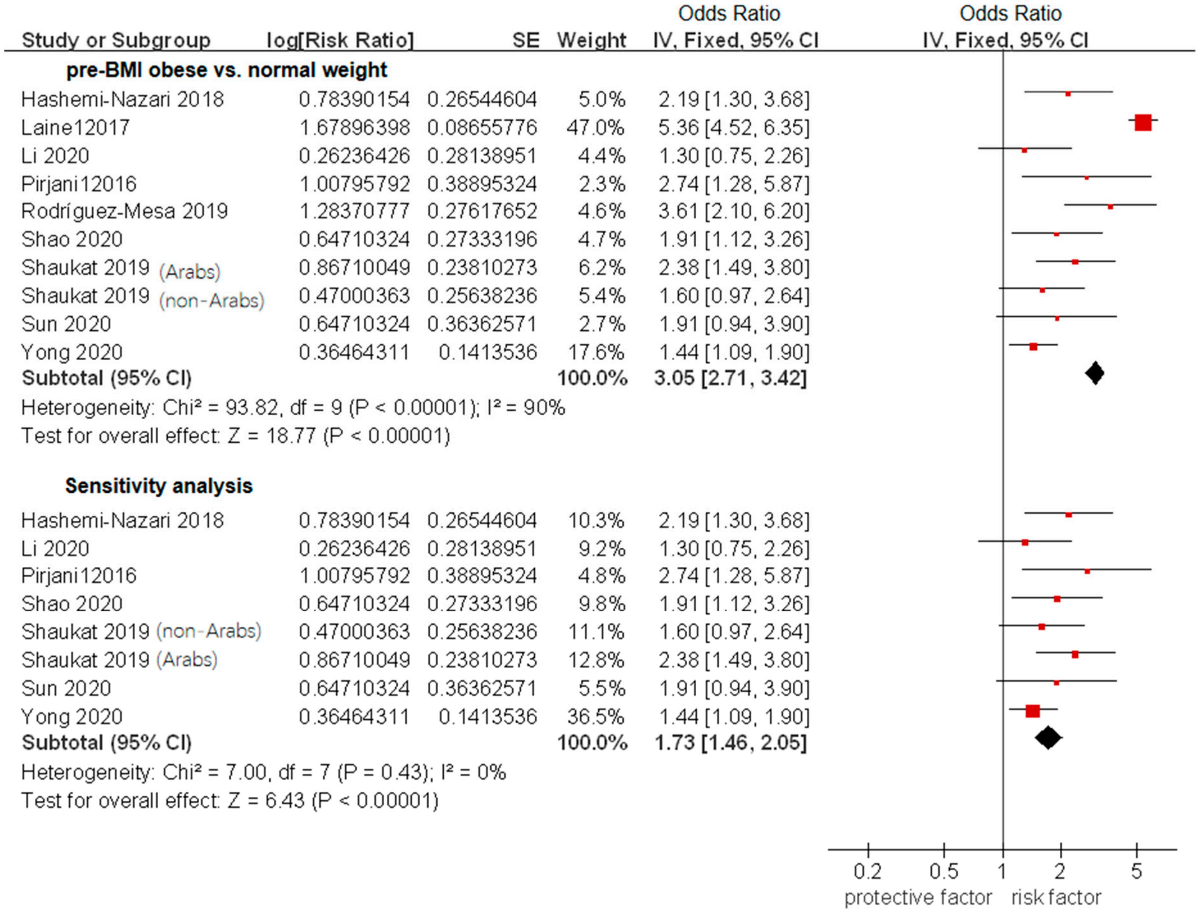

Supplementary Figure S5. Age at menarche

Study or Subgroup	log[Odds Ratio]	SE	Weight	Odds Ratio IV, Random, 95% CI
**Age at menarche ≤ 11 yr vs. > 11 yr**				
Chen 2015	0.29266961	0.08326444	42.4%	1.34 [1.14, 1.58]
Dishi 2011	-0.19845094	0.24259599	14.6%	0.82 [0.51, 1.32]
Li 2016	0.3435897	0.14481365	28.0%	1.41 [1.06, 1.87]
Shen 2016	0.55961579	0.23743148	15.1%	1.75 [1.10, 2.79]
**Subtotal (95% CI)**			**100.0%**	**1.32 [1.07, 1.63]**

Heterogeneity: Tau² = 0.02; Chi² = 5.48, df = 3 (P = 0.14); I² = 45%

Test for overall effect: Z = 2.56 (P = 0.01)

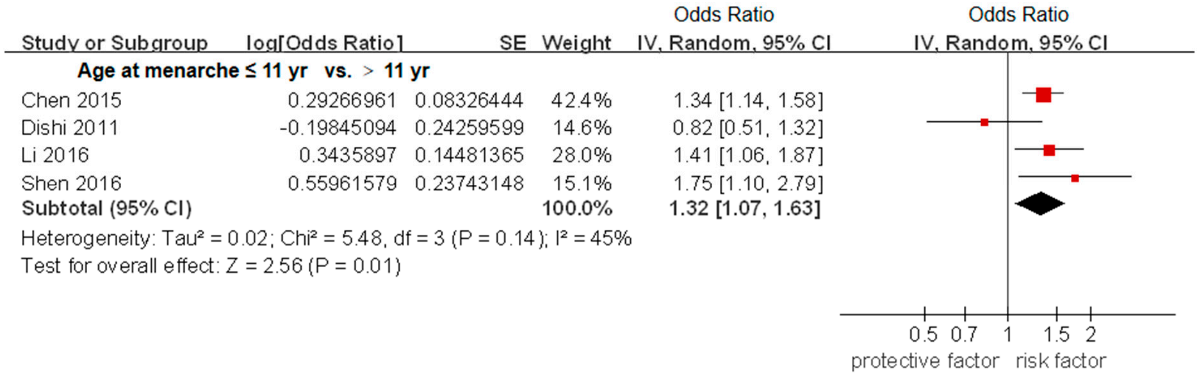

Supplementary Figure S6. Acceptance for ART treatment

Study or Subgroup	log[Odds Ratio]	SE	Weight	Odds Ratio IV, Random, 95% CI
**ART vs. natural conception**				
Barua 2016	0.41144718	0.0759752	8.2%	1.51 [1.30, 1.75]
Hu2019 (fresh cycles)	0.32353172	0.1622122	5.8%	1.38 [1.01, 1.90]
Hu2019 (secondary infertility)	0.17142912	0.16922585	5.6%	1.19 [0.85, 1.65]
Hu2019 (ICSI)	-0.15198636	0.19047957	5.1%	0.86 [0.59, 1.25]
Lei 2019	0.61518564	0.20595397	4.7%	1.85 [1.24, 2.77]
Nagata 2019 (ICSI)	-0.03045921	0.10130022	7.5%	0.97 [0.80, 1.18]
Nagata 2019 (IVT-ET)	-0.01005034	0.14227276	6.3%	0.99 [0.75, 1.31]
Nagata 2019 (ovulation induction)	-0.03045921	0.13778622	6.5%	0.97 [0.74, 1.27]
Shevell2005 (IVF)	-0.69314718	0.4105709	1.9%	0.50 [0.22, 1.12]
Shevell2005 (ovulation induction)	0.40546511	0.17682326	5.4%	1.50 [1.06, 2.12]
Silberstein 2013	0.57097955	0.07878527	8.1%	1.77 [1.52, 2.07]
Stern 2014 (tubal)	0.35065687	0.13356834	6.6%	1.42 [1.09, 1.84]
Stern 2014 (ovulation disorders)	0.77472717	0.11785136	7.0%	2.17 [1.72, 2.73]
Stern 2014 (male factor)	0.13976194	0.09257793	7.7%	1.15 [0.96, 1.38]
Stern 2014 (endometriosis)	-0.07257069	0.20595397	4.7%	0.93 [0.62, 1.39]
Wang 2013	0.23111172	0.0362169	8.9%	1.26 [1.17, 1.35]
**Subtotal (95% CI)**			**100.0%**	**1.27 [1.12, 1.44]**
Heterogeneity: Tau² = 0.04; Chi² = 74.34, df = 15 (P < 0.00001); I² = 80%				
Test for overall effect: Z = 3.73 (P = 0.0002)				
**Sensitivity analysis**				
Barua 2016	0.41144718	0.0759752	11.0%	1.51 [1.30, 1.75]
Hu2019 (fresh cycles)	-0.15198636	0.19047957	5.2%	0.86 [0.59, 1.25]
Hu2019 (secondary infertility)	0.17142912	0.16922585	6.0%	1.19 [0.85, 1.65]
Hu2019 (ICSI)	0.32353172	0.1622122	6.3%	1.38 [1.01, 1.90]
Lei 2019	0.61518564	0.20595397	4.7%	1.85 [1.24, 2.77]
Nagata 2019 (ICSI)	-0.03045921	0.10130022	9.4%	0.97 [0.80, 1.18]
Nagata 2019 (IVT-ET)	-0.01005034	0.14227276	7.2%	0.99 [0.75, 1.31]
Nagata 2019 (ovulation induction)	-0.03045921	0.13778622	7.4%	0.97 [0.74, 1.27]
Shevell2005 (IVF)	0.40546511	0.17682326	5.7%	1.50 [1.06, 2.12]
Shevell2005 (ovulation induction)	-0.69314718	0.4105709	1.6%	0.50 [0.22, 1.12]
Stern 2014 (male factor)	0.13976194	0.09257793	10.0%	1.15 [0.96, 1.38]
Stern 2014 (endometriosis)	-0.07257069	0.20595397	4.7%	0.93 [0.62, 1.39]
Stern 2014 (tubal)	0.35065687	0.13356834	7.6%	1.42 [1.09, 1.84]
Wang 2013	0.23111172	0.0362169	13.1%	1.26 [1.17, 1.35]
**Subtotal (95% CI)**			**100.0%**	**1.19 [1.06, 1.32]**
Heterogeneity: Tau² = 0.02; Chi² = 36.09, df = 13 (P = 0.0006); I² = 64%				
Test for overall effect: Z = 3.10 (P = 0.002)				

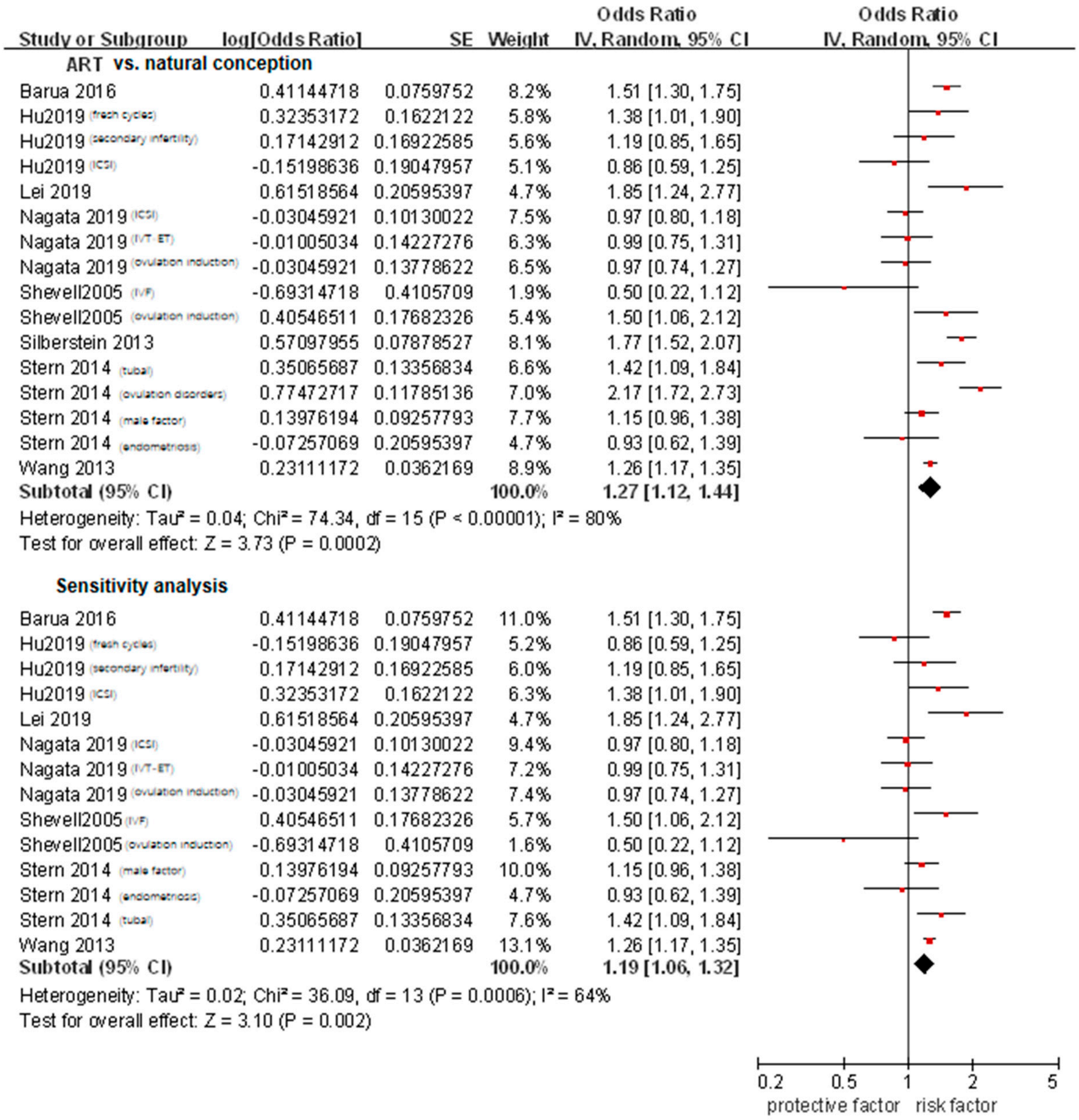

Supplementary Figure S7. Positive status for thyroid-related antibodies

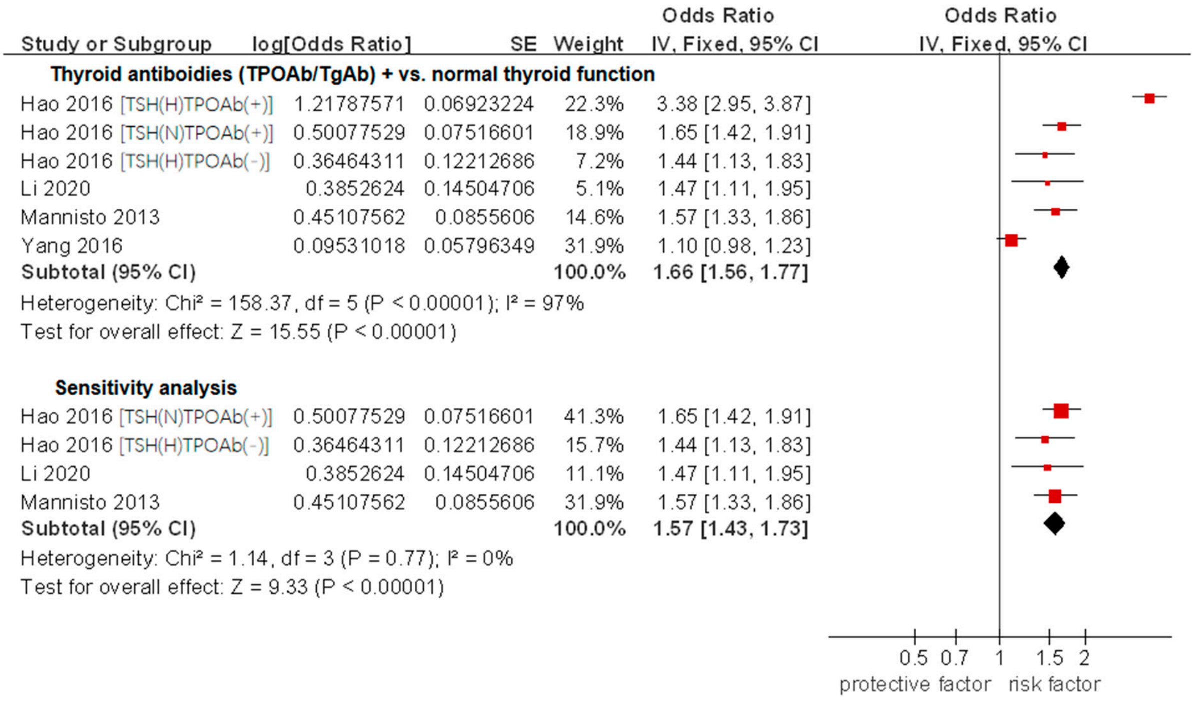

Supplementary Figure S8. GWG at the 1st trimester

Study or Subgroup	log[Odds Ratio]	SE	Weight	Odds Ratio IV, Random, 95% CI	Odds Ratio IV, Random, 95% CI
**Above IOM recommended GWG at the 1st trimester vs. normal GWG**					
Hedderson 2010	0.42526774	0.1732883	26.7%	1.53 [1.09, 2.15]	
Qi 2020	0.06765865	0.06441218	47.5%	1.07 [0.94, 1.21]	
Zhong 2017	0.33647224	0.17941315	25.8%	1.40 [0.98, 1.99]	
**Subtotal (95% CI)**			**100.0%**	**1.26 [0.99, 1.61]**	

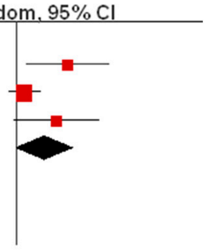

Heterogeneity: Tau² = 0.03; Chi² = 5.16, df = 2 (P = 0.08); I² = 61%
Test for overall effect: Z = 1.85 (P = 0.06)

Supplementary Figure S9. Funnel plot of the included variables in the score-scaled GDM prediction model

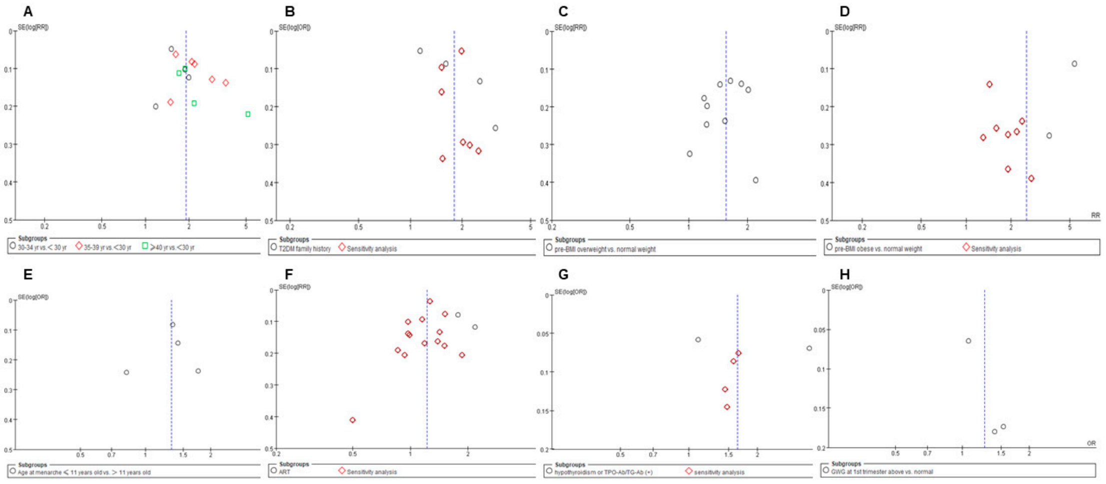

Funnel plot of age **(A)**, T2DM family history **(B)**, pre-BMI overweight **(C)**, pre-BMI obese **(D)**, age at menarche **(E)**, accept ART treatment **(F)**, thyroid relative antibodies positive **(G)**, GWG at the 1st trimester **(H)**

Supplementary Figure S10. The ROC curves for the two ML models

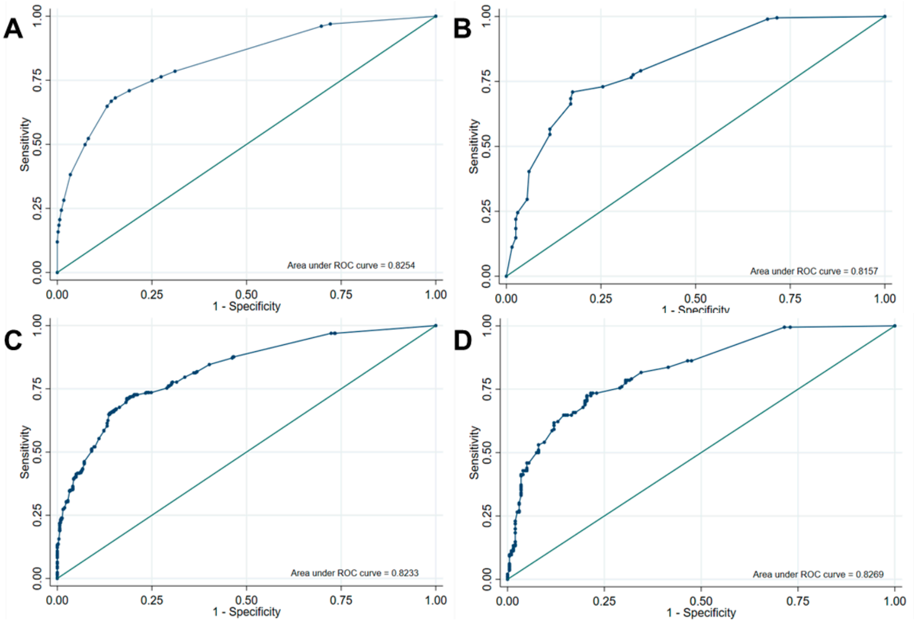

(A) ROC curve of the training set for the decision tree model and **(B)** the ROC curve for the validation set. **(C)** ROC curve for the training set for the random forest model, and **(D)** the ROC curve for the validation set.

Supplementary Figure S11. The tree structure in the decision tree model

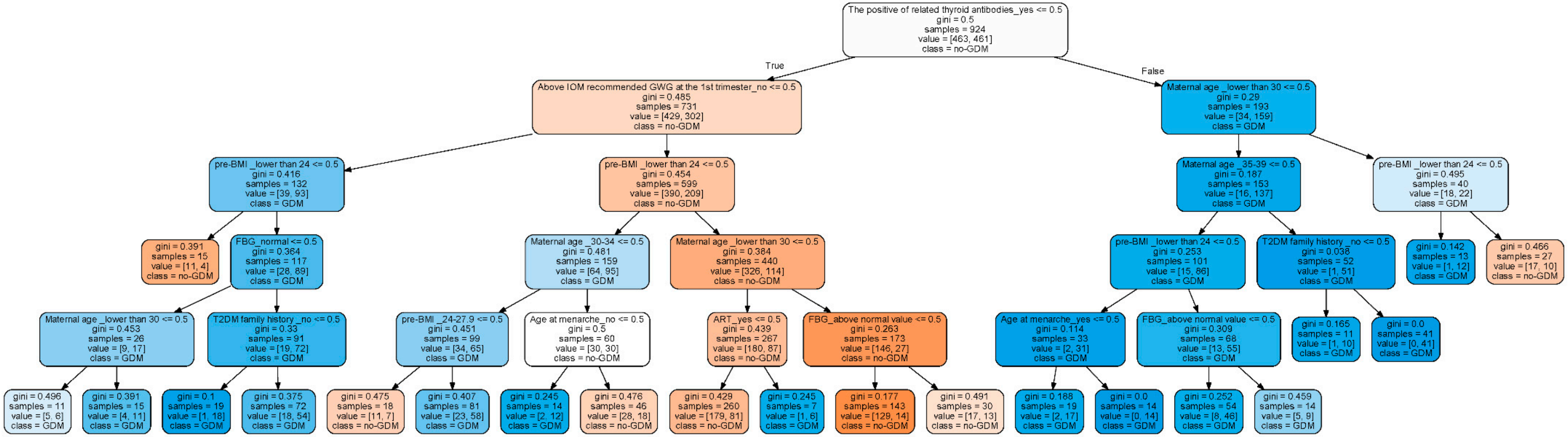

Supplementary Figure S12. The tree structure in the random forest model

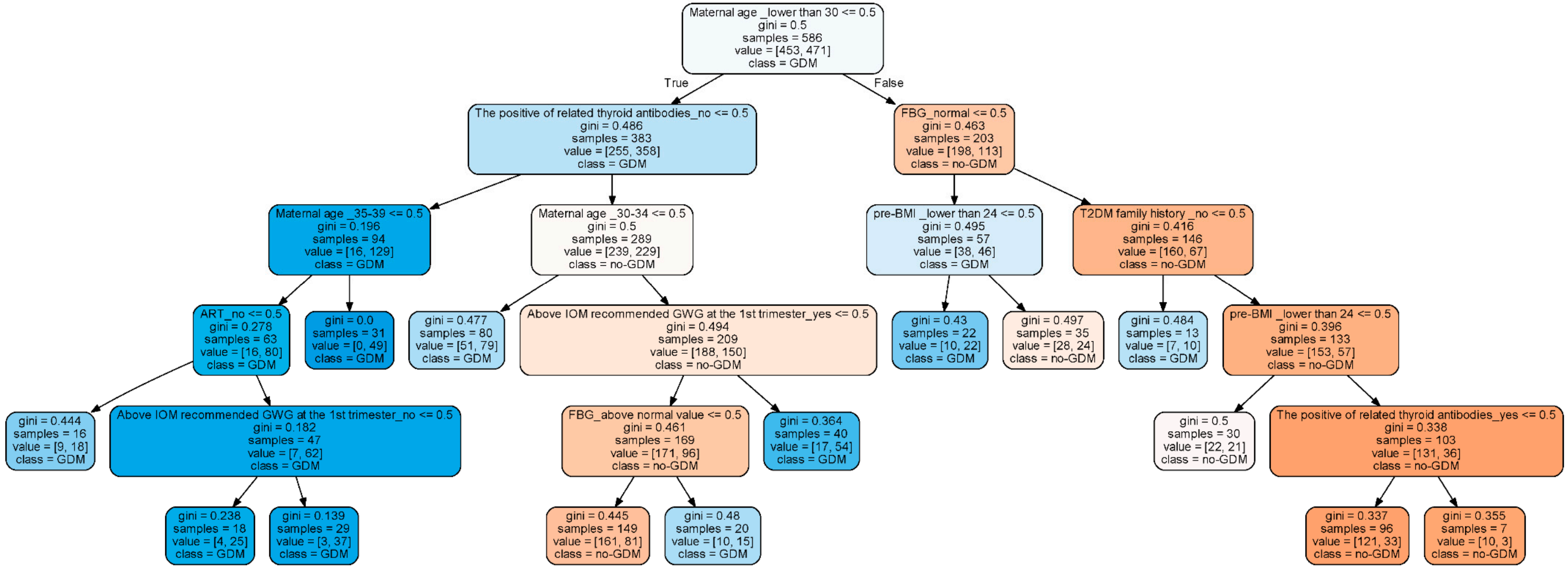

Supplementary Figure S13. Feature importance curve derived from the decision tree and random forest models

A

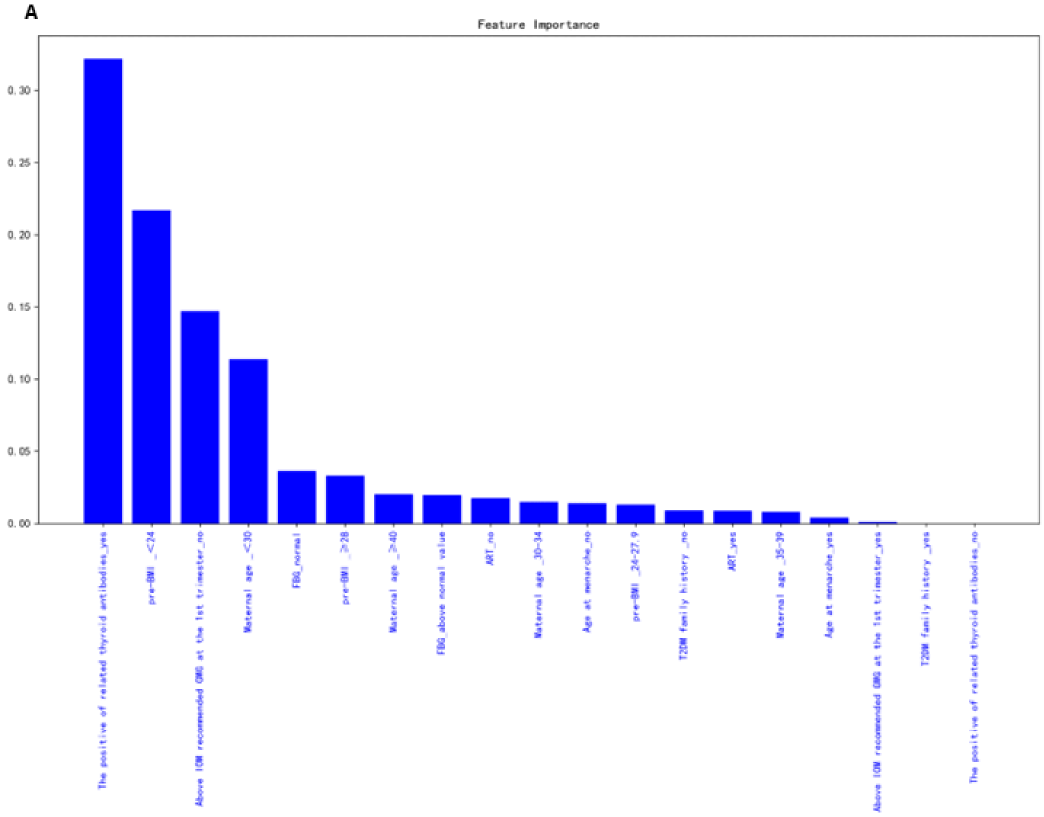

B

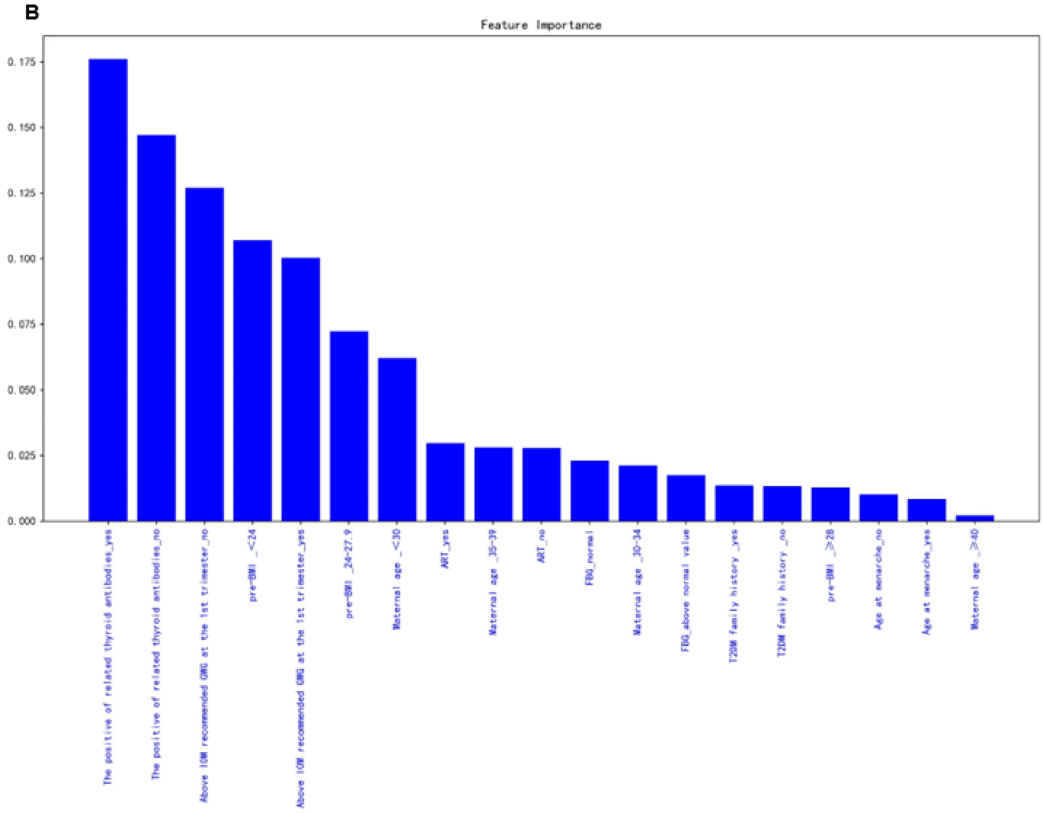

(A) Decision tree model. **(B)** Random forest model.

Studies included in the meta-analysis

1. Qi, Y. N.; Sun, X.; Tan, J.; Zhang, G. T.; Chen, M.; Xiong, Y. Q.; Chen, P.; Liu, C. R.; Zou, K.; Liu, X. H., Excessive gestational weight gain in the first and second trimester is a risk factor for gestational diabetes mellitus among women pregnant with singletons: A repeated measures analysis. *J Diabetes Invest* **2020,** *11* (6), 1651-1660.
2. Hedderson, M. M.; Gunderson, E. P.; Ferrara, A., Gestational Weight Gain and Risk of Gestational Diabetes Mellitus. *Obstet Gynecol* **2010,** *115* (3), 597-604.
3. Zhong, C. R.; Li, X. T.; Chen, R. J.; Zhou, X. Z.; Liu, C. Q.; Wu, J. Y.; Xu, S. Z.; Wang, W. Y.; Xiao, M.; Xiong, G. P.; Wang, J.; Yang, X. F.; Hao, L. P.; Yang, N. H., Greater early and mid-pregnancy gestational weight gain are associated with increased risk of gestational diabetes mellitus: A prospective cohort study. *Clin Nutr Espen* **2017,** *22*, 48-53.
4. Chen, L. W.; Li, S. S.; He, C. Y.; Zhu, Y. Y.; Louis, G. M. B.; Yeung, E.; Hu, F. B.; Zhang, C. L., Age at Menarche and Risk of Gestational Diabetes Mellitus: A Prospective Cohort Study Among 27,482 Women. *Diabetes Care* **2016,** *39* (3), 469-471.
5. Dishi, M.; Enquobahrie, D. A.; Abetew, D. F.; Qiu, C. F.; Rudra, C. B.; Williams, M. A., Age at menarche, menstrual cycle characteristics and risk of gestational diabetes. *Diabetes Res Clin Pr* **2011,** *93* (3), 437-442.
6. Li, H.; Shen, L.; Song, L.; Liu, B.; Zheng, X.; Xu, S.; Wang, Y., Early age at menarche and gestational diabetes mellitus risk: Results from the Healthy Baby Cohort study. *Diabetes Metab* **2017,** *43* (3), 248-252.
7. Shen, Y.; Hu, H.; Taylor, B. D.; Kan, H. D.; Xu, X. H., Early Menarche and Gestational Diabetes Mellitus at First Live Birth. *Matern Child Hlth J* **2017,** *21* (3), 593-598.
8. Ying, H.; Tang, Y. P.; Bao, Y. R.; Su, X. J.; Cai, X.; Li, Y. H.; Wang, D. F., Maternal TSH level and TPOAb status in early pregnancy and their relationship to the risk of gestational diabetes mellitus. *Endocrine* **2016,** *54* (3), 742-750.
9. Li, G. J.; Wei, T.; Ni, W.; Zhang, A.; Zhang, J.; Xing, Y. H.; Xing, Q. S., Incidence and Risk Factors of Gestational Diabetes Mellitus: A Prospective Cohort Study in Qingdao, China. *Front Endocrinol* **2020,** *11*.
10. Mannisto, T.; Mendola, P.; Grewal, J.; Xie, Y. L.; Chen, Z.; Laughon, S. K., Thyroid Diseases and Adverse Pregnancy Outcomes in a Contemporary US Cohort. *J Clin Endocr Metab* **2013,** *98* (7), 2725-2733.
11. Yang, S.; Shi, F. T.; Leung, P. C. K.; Huang, H. F.; Fan, J. X., Low Thyroid Hormone in Early Pregnancy Is Associated With an Increased Risk of Gestational Diabetes Mellitus. *J Clin Endocr Metab* **2016,** *101* (11), 4237-4243.
12. Lei, L. L.; Lan, Y. L.; Wang, S. Y.; Feng, W.; Zhai, Z. J., Perinatal complications and live-birth outcomes following assisted reproductive technology: a retrospective cohort study. *Chinese Med J-Peking* **2019,** *132* (20), 2408-2416.
13. Barua, S.; Hng, T. M.; Smith, H.; Bradford, J.; McLean, M., Ovulatory disorders are an independent risk factor for pregnancy complications in women receiving assisted reproduction treatments. *Aust Nz J Obstet Gyn* **2017,** *57* (3), 286-293.
14. Hu, S. Q.; Xu, B.; Zhang, Y. N.; Jin, L., Risk factors of gestational diabetes mellitus during assisted reproductive technology procedures. *Gynecol Endocrinol* **2020,** *36* (4), 318-321.
15. Nagata, C.; Yang, L. M.; Yamamoto-Hanada, K.; Mezawa, H.; Ayabe, T.; Ishizuka, K.; Konishi, M.; Ohya, Y.; Saito, H.; Sago, H.; Kawamoto, T.; Kishi, R.; Yaegashi, N.;

Hashimoto, K.; Mori, C.; Ito, S.; Yamagata, Z.; Inadera, H.; Kamijima, M.; Nakayama, T.; Iso, H.; Shima, M.; Hirooka, Y.; Suganuma, N.; Kusuhara, K.; Katoh, T.; Study, J. E. C., Complications and adverse outcomes in pregnancy and childbirth among women who conceived by assisted reproductive technologies: a nationwide birth cohort study of Japan environment and children's study. *Bmc Pregnancy Childb* **2019,** *19*.
16. Shevell, T.; Malone, F. D.; Vidaver, J.; Porter, T. F.; Luthy, D. A.; Comstock, C. H.; Hankins, G. D.; Eddleman, K.; Dolan, S.; Dugoff, L.; Craigo, S. D.; Timor, I. E.; Carr, S. R.; Wolfe, H. M.; D'Alton, M. E., Assisted reproductive technology and pregnancy outcome - A population based screening study (the FASTER trial). *Am J Obstet Gynecol* **2003,** *189* (6), S175-S175.
17. Silberstein, T.; Levy, A.; Harlev, A.; Saphier, O.; Sheiner, E., Perinatal outcome of pregnancies following in vitro fertilization and ovulation induction. *J Matern-Fetal Neo M* **2014,** *27* (13), 1316-1319.
18. Stern, J. E.; Luke, B.; Tobias, M.; Gopal, D.; Hornstein, M. D.; Diop, H., Adverse pregnancy and birth outcomes associated with underlying diagnosis with and without assisted reproductive technology treatment. *Fertil Steril* **2015,** *103* (6), 1438-1445.
19. Wang, Y. A.; Nikravan, R.; Smith, H. C.; Sullivan, E. A., Higher prevalence of gestational diabetes mellitus following assisted reproduction technology treatment. *Hum Reprod* **2013,** *28* (9), 2554-2561.
20. Cosson, E.; Cussac-Pillegand, C.; Benbara, A.; Pharisien, I.; Jaber, Y.; Banu, I.; Nguyen, M. T.; Valensi, P.; Carbillon, L., The Diagnostic and Prognostic Performance of a Selective Screening Strategy for Gestational Diabetes Mellitus According to Ethnicity in Europe. *J Clin Endocr Metab* **2014,** *99* (3), 996-1005.
21. Schoenaker, D. A. J. M.; Vergouwe, Y.; Soedamah-Muthu, S. S.; Callaway, L. K.; Mishra, G. D., Preconception risk of gestational diabetes: Development of a prediction model in nulliparous Australian women. *Diabetes Res Clin Pr* **2018,** *146*, 48-57.
22. Hosseini, E.; Janghorbani, M.; Shahshahan, Z., Comparison of risk factors and pregnancy outcomes of gestational diabetes mellitus diagnosed during early and late pregnancy. *Midwifery* **2018,** *66*, 64-69.
23. Larrabure-Torrealva, G. T.; Martinez, S.; Luque-Fernandez, M. A.; Sanchez, S. E.; Mascaro, P. A.; Ingar, H.; Castillo, W.; Zumaeta, R.; Grande, M.; Motta, V.; Pacora, P.; Gelaye, B.; Williams, M. A., Prevalence and risk factors of gestational diabetes mellitus: findings from a universal screening feasibility program in Lima, Peru. *Bmc Pregnancy Childb* **2018,** *18*.
24. Leng, J. H.; Shao, P.; Zhang, C. P.; Tian, H. G.; Zhang, F. X.; Zhang, S.; Dong, L.; Li, L. L.; Yu, Z. J.; Chan, J. C. N.; Hu, G.; Yang, X. L., Prevalence of Gestational Diabetes Mellitus and Its Risk Factors in Chinese Pregnant Women: A Prospective Population-Based Study in Tianjin, China. *Plos One* **2015,** *10* (3).
25. Pirjani, R.; Shirzad, N.; Qorbani, M.; Phelpheli, M.; Nasli-Esfahani, E.; Bandarian, F.; Hemmatabadi, M., Gestational diabetes mellitus its association with obesity: a prospective cohort study. *Eat Weight Disord-St* **2017,** *22* (3), 445-450.
26. Schaefer, K. K.; Xiao, W. Q.; Chen, Q. Z.; He, J. R.; Lu, J. H.; Chan, F. F.; Chen, N. N.; Yuan, M. Y.; Xia, H. M.; Lam, K. B. H.; Hirst, J. E.; Qiu, X., Prediction of gestational diabetes mellitus in the Born in Guangzhou Cohort Study, China. *Int J Gynecol Obstet* **2018,** *143* (2), 164-171.

27. Shahbazian, H.; Nouhjah, S.; Shahbazian, N.; Jahanfar, S.; Latifi, S. M.; Aleali, A.; Shahbazian, N.; Saadati, N., Gestational diabetes mellitus in an Iranian pregnant population using IADPSG criteria: Incidence, contributing factors and outcomes. *Diabetes Metab Syndr* **2016,** *10* (4), 242-246.
28. Wang, Y.; Luo, B., Risk factors analysis of gestational diabetes mellitus based on International Association of Diabetes Pregnancy Study Groups criteria. *Nan fang yi ke da xue xue bao = Journal of Southern Medical University* **2019,** *39* (5), 572-578.
29. Yan, B.; Yu, Y. X.; Lin, M. Z.; Li, Z. B.; Wang, L. Y.; Huang, P. Y.; Song, H. Q.; Shi, X. L.; Yang, S. Y.; Li, X. Y.; Li, X. J., High, but stable, trend in the prevalence of gestational diabetes mellitus: A population-based study in Xiamen, China. *J Diabetes Invest* **2019,** *10* (5), 1358-1364.
30. Khalil, A.; Syngelaki, A.; Maiz, N.; Zinevich, Y.; Nicolaides, K. H., Maternal Age and Adverse Pregnancy Outcomes: A Cohort Study EDITORIAL COMMENT. *Obstet Gynecol Surv* **2013,** *68* (12), 779-781.
31. Londero, A. P.; Rossetti, E.; Pittini, C.; Cagnacci, A.; Driul, L., Maternal age and the risk of adverse pregnancy outcomes: a retrospective cohort study. *Bmc Pregnancy Childb* **2019,** *19*.
32. Sweeting, A. N.; Appelblom, H.; Ross, G. P.; Wong, J.; Kouru, H.; Williams, P. F.; Sairanen, M.; Hyett, J. A., First trimester prediction of gestational diabetes mellitus: A clinical model based on maternal demographic parameters. *Diabetes Res Clin Pr* **2017,** *127*, 44-50.
33. Wang, C.; Wang, X. Y.; Yang, H. X., Effect of maternal age on pregnancy outcomes in Beijing. *Zhonghua fu chan ke za zhi* **2017,** *52* (8), 514-520.
34. Koo, Y. J.; Ryu, H. M.; Yang, J. H.; Lim, J. H.; Lee, J. E.; Kim, M. Y.; Chung, J. H., Pregnancy outcomes according to increasing maternal age. *Taiwan J Obstet Gyne* **2012,** *51* (1), 60-65.
35. Sun, Y.; Shen, Z. Z.; Zhan, Y. L.; Wang, Y. W.; Ma, S.; Zhang, S. H.; Liu, J. T.; Wu, S. S.; Feng, Y. H.; Chen, Y. L.; Cai, S. Y.; Shi, Y. J.; Ma, L. K.; Jiang, Y., Effects of pre-pregnancy body mass index and gestational weight gain on maternal and infant complications. *Bmc Pregnancy Childb* **2020,** *20* (1).
36. Rodriguez-Mesa, N.; Robles-Benayas, P.; Rodriguez-Lopez, Y.; Perez-Fernandez, E. M.; Cobo-Cuenca, A. I., Influence of Body Mass Index on Gestation and Delivery in Nulliparous Women: A Cohort Study. *Int J Env Res Pub He* **2019,** *16* (11).
37. Shaukat, S.; Nur, U., Effect of prepregnancy maternal BMI on adverse pregnancy and neonatal outcomes: results from a retrospective cohort study of a multiethnic population in Qatar. *Bmj Open* **2019,** *9* (9).
38. Gao, X. A.; Yan, Y.; Xiang, S. T.; Zeng, G. Y.; Liu, S. P.; Sha, T. T.; He, Q. O.; Li, H. Y.; Tan, S.; Chen, C.; Li, L.; Yan, Q. A., The mutual effect of pre-pregnancy body mass index, waist circumference and gestational weight gain on obesity-related adverse pregnancy outcomes: A birth cohort study. *Plos One* **2017,** *12* (6).
39. Laine, M. K.; Kautiainen, H.; Gissler, M.; Raina, M.; Aahos, I.; Jarvinen, K.; Pennanen, P.; Eriksson, J. G., Gestational diabetes in primiparous women-impact of age and adiposity: a register-based cohort study. *Acta Obstet Gyn Scan* **2018,** *97* (2), 187-194.
40. Yong, H. Y.; Shariff, Z. M.; Yusof, B. N. M.; Rejali, Z.; Tee, Y. Y. S.; Bindels, J.; van Der Beek, E. M., Independent and combined effects of age, body mass index and gestational weight gain on the risk of gestational diabetes mellitus. *Sci Rep-Uk* **2020,** *10* (1).
41. Hashemi-Nazari, S. S.; Najafi, F.; Rahimi, M. A.; Izadi, N.; Heydarpour, F.; Forooghirad,

H., Estimation of gestational diabetes mellitus and dose-response association of BMI with the occurrence of diabetes mellitus in pregnant women of the west of Iran. *Health Care Women In* **2020,** *41* (1), 121-130.
42. Shao, B. L.; Mo, M. J.; Xin, X.; Jiang, W.; Wu, J. H.; Huang, M. X.; Wang, S. J.; Muyiduli, X.; Si, S. T.; Shen, Y.; Chen, Z. X.; Yu, Y. X., The interaction between prepregnancy BMI and gestational vitamin D deficiency on the risk of gestational diabetes mellitus subtypes with elevated fasting blood glucose. *Clin Nutr* **2020,** *39* (7), 2265-2273.
